# Chemotherapy‐Enriched THBS2‐Deficient Cancer Stem Cells Drive Hepatocarcinogenesis through Matrix Softness Induced Histone H3 Modifications

**DOI:** 10.1002/advs.202002483

**Published:** 2021-01-04

**Authors:** Kai‐Yu Ng, Queenie T. Shea, Tin‐Lok Wong, Steve T. Luk, Man Tong, Chung‐Mau Lo, Kwan Man, Jing‐Ping Yun, Xin‐Yuan Guan, Terence K. Lee, Yong‐Ping Zheng, Stephanie Ma

**Affiliations:** ^1^ School of Biomedical Sciences Li Ka Shing Faculty of Medicine The University of Hong Kong Pokfulam Hong Kong; ^2^ Department of Biomedical Engineering The Hong Kong Polytechnic University Hung Hom Kowloon Hong Kong; ^3^ State Key Laboratory of Liver Research The University of Hong Kong Pokfulam Hong Kong; ^4^ Department of Surgery Queen Mary Hospital The University of Hong Kong Pokfulam Hong Kong; ^5^ The University of Hong Kong ‐ Shenzhen Hospital Shenzhen Guangdong 518009 China; ^6^ Department of Pathology Sun Yat‐Sen University Cancer Centre Guangzhou Guangdong 510060 China; ^7^ Department of Clinical Oncology Queen Mary Hospital The University of Hong Kong Pokfulam Hong Kong; ^8^ Department of Applied Biology and Chemical Technology The Hong Kong Polytechnic University Hung Hom Kowloon Hong Kong

**Keywords:** cancer stemness, CD133, hepatocellular carcinomas, histone modifications, matrix stiffness, mechanoepigenetics, metastasis, THBS2

## Abstract

The physical microenvironment is a critical mediator of tumor behavior. However, detailed biological and mechanistic insight is lacking. The present study reveals the role of chemotherapy‐enriched CD133+ liver cancer stem cells (CSCs) with THBS2 deficiency. This subpopulation of cells contributes to a more aggressive cancer and functional stemness phenotype in hepatocellular carcinoma (HCC) by remodeling the extracellular matrix (ECM) through the regulation of matrix metalloproteinase (MMP) activity, collagen degradation, and matrix stiffness. The local soft spots created by these liver CSCs can enhance stemness and drug resistance and provide a route of escape to facilitate HCC metastasis. Interestingly, a positive feed‐forward loop is identified where a local soft spot microenvironment in the HCC tumor is enriched with CD133 expressing cells that secrete markedly less ECM‐modifying THBS2 upon histone H3 modification at its promoter region, allowing the maintenance of a localized soft spot matrix. Clinically, THBS2 deficiency is also correlated with low HCC survival, where high levels of CSCs with low THBS2 expression in HCC are associated with decreased collagen fiber deposits and an invasive tumor front. The findings have implications for the treatment of cancer stemness and for the prevention of tumor outgrowth through disseminated tumor cells.

## Graphical abstract

Chemotherapy enrichment for CD133 expressing cells with deficient THBS2 expression, which facilitates the promotion of cancer stemness properties in hepatocellular carcinoma (HCC) cells through extracellular matrix remodeling, is described. The resultant local soft microenvironment induces mechanoepigenetic changes through histone H3 modifications, facilitating a positive feedback loop that supports the clonogenic expansion of a HCC cells with high CD133 and low THBS2 expression.

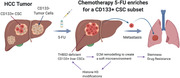

## 1 Introduction

There is now accumulating evidence to suggest that a “stem cell niche” is of paramount importance for the maintenance of the self‐renewing capacity and undifferentiated state of stem cells.^[^ ^1^ ^]^ The same notion of a niche is applicable to cancer stem cells (CSCs), a subpopulation of tumor cells that is now widely accepted to be associated with drug resistance, heterogeneity, tumor relapse, and the general unfavorable outcome of cancer patients. Components residing in this niche that help CSCs maintain their stemness include various epithelial and nonepithelial cell types and the plethora of factors they secrete as well as reactive oxygen species, tissue hypoxia, and extracellular matrix (ECM) remodeling.^[^ ^2^ ^]^ Additionally, in recent years, physical properties of the microenvironment including their tension and stiffness are also progressively recognized as critical players in carcinogenesis.^[^ ^3^, ^4^ ^]^ Past studies have shown that different biomechanical components including diverse matrix stiffnesses can contribute to the plasticity of CSCs.^[^ ^5^ ^]^ Matrix stiffness can enhance functional stemness phenotype in breast and colon cancers through altering integrin‐linked kinase^[^ ^6^, ^7^ ^]^ and Yes‐associated protein,^[^ ^8^ ^]^ respectively. Remarkably, in vitro studies and clinical data have indicated that cancerous tissue displays not only biological, but also mechanical heterogeneity. For instance, stiffness of the core and invasive front is different,^[^ ^9^, ^10^ ^]^ while distribution of CSCs in the tumor tissue is not uniform. In line with these findings, some glioma stem cells have been shown to be preferentially located at the invasive front rather than at the core in glioma tissues.^[^ ^11^ ^]^

Hepatocellular carcinoma (HCC), the main type of liver cancer in human, is one of the most prevalent malignancies globally. The disease is unique in that more than 80% of HCC patients develop in the setting of liver fibrosis and cirrhosis. Increased liver stiffness is long recognized as an important risk factor for HCC progression. Liver fibrosis is characterized by alterations in both the biochemical and physical properties of the cellular microenvironment. However, whether mechanical factors play a role in modulating hepatocarcinogenesis remains poorly understood. Ultrasound elastography like FibroScan have demonstrated that liver stiffness measurements are a reliable predictor of HCC growth.^[^ ^12^, ^13^ ^]^ Thus, there is sound evidence to show that HCC develops in a niche with mechanical properties unlike those encountered in the normal liver. Despite definite improvements in the outcome of patients with HCC, the overall prognosis of this cancer is still unsatisfactory because of late presentation and frequent tumor recurrence. HCC remains difficult to treat, owing to a paucity of drugs that target critical dependencies; and the fact that FDA approved chemotherapy and broad‐spectrum kinase inhibitors such as sorafenib, lenvatinib, and the more recently approved combination of atezolizumab and bevacizumab provide only a modest benefit to patients with the disease. Tumor relapse and drug resistances, which have been extensively shown to be attributed to the existence of CSCs, also remain major obstacles in the treatment of the disease.^[^ ^14^, ^15^ ^]^ In the context of HCC, the link between matrix stiffness and stemness has remained very controversial. Low‐level shear stress has been found to induce differentiation of liver CSCs via the Wnt/*β*‐catenin signaling pathway.^[^ ^16^ ^]^ Matrix stiffness has also been found to enhance CD133+/EpCAM+ expression and functional stemness by activating the integrin *β*1/AKT/mTOR/SOX2 signaling cascade.^[^ ^17^ ^]^ However, Schrader et al. found that surviving chemotherapy‐enriched cells grown on soft supports had markedly enhanced clonogenic ability than surviving cells grown in a stiff environment and displayed elevated expression of CSC markers, including CD133, CD44, NANOG, CXCR4, and c‐kit.^[^ ^18^ ^]^ More recently, Tian et al. found that a soft matrix enhanced the expression of liver CSC markers (CD133 and CD90) and the number of HCC cells with a functional CSC phenotype.^[^ ^19^ ^]^ Studies to date have, however, been limited to 2D culture experimental setups in vitro, with few in vivo elastography stiffness measurements. The mechanism explaining how altered matrix stiffness/softness alters stemness characteristics has also not been elucidated.

Our present study discovered the role of chemotherapy‐enriched CD133+ liver CSCs with THBS2 deficiency. This subpopulation of cells contributes to cancer aggressiveness and a functional stemness phenotype in HCC cells by remodeling the ECM by regulating matrix metalloproteinase (MMP) activity, collagen degradation, and matrix stiffness. The local soft spot created by these liver CSCs can enhance stemness and drug resistance and provides a route of cell escape that facilitates HCC metastasis. Interestingly, we identified a positive feed‐forward loop where a local soft spot microenvironment in the HCC tumor is enriched for CD133 expressing cells that secretes markedly less ECM‐modifying THBS2 because of histone H3 modification at the promoter region, allowing the maintenance of a local matrix soft spot. These findings are substantiated in vitro and in vivo using various disease models, including patient‐derived HCC organoids and HCC cell lines grown on a 3D matrix, immunodeficient and immunocompetent HCC mouse models. Our observations in human clinical tissue samples suggest that the distribution of THBS2‐deficient CD133+ liver CSCs in HCC tissue is not uniform and is maintained in a local soft environment toward the invasive tumor front. Our findings have implications for the treatment of cancer stemness and for the prevention of tumor outgrowth through disseminated tumor cells.

## 2 Results

### 2.1 Chemotherapy‐enriched THBS2‐deficient CD133+ liver cancer stem cells confer an enhanced ability to degrade the ECM by modulating MMP activity

**Figure 1:**
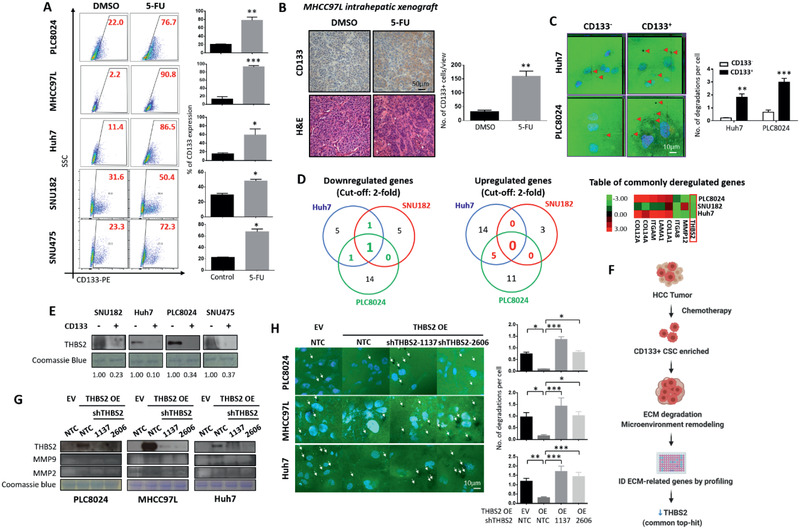
Chemotherapy‐enriched THBS2‐deficient CD133+ liver cancer stem cells confer an enhanced ability to degrade the ECM by modulating MMP activity. A) Representative flow cytometry dot plots and quantification of CD133 expression in HCC cells with or without 5‐fluorouracil (5‐FU) treatment. DMSO was used as a control. B) Representative H&E and immunohistochemical staining for CD133 expression in orthotopic liver MHCC97L xenograft tumors with or without 5‐FU treatment. Bar graph shows quantitation of number of CD133+ cells. DMSO was used as a control. Scale bar: 50 µm. C) Representative images and quantification of gelatin degradation assays of sorted CD133+ cancer stem cell versus CD133− noncancer stem cell subpopulations in HCC cells. Red arrows point to the area of degraded gelatin. Scale bar: 10 µm. D) PCR array profiles of genes related to extracellular matrix and adhesion molecules. Venn diagrams (top) showing the number of downregulated (3) and upregulated genes (5) in CD133+ versus CD133− in 2 of 3 subpopulation sorted by HCC cell type (PLC8024, SNU182, and Huh7). Heatmap (bottom) of commonly dysregulated genes as analyzed by PCR array. Expression values were normalized to the expression of 5 housekeeping genes and presented as fold changes in CD133+ to CD133− subpopulations. E) Western blot validation of secreted THBS2 proteomic expression in sorted CD133 subpopulation of HCC cells. Coomassie blue was used as the loading control. F) Image showing the rationale used for THBS2 identification. G) Gelatin zymography for MMP2 and MMP9 activities and H) representative images and quantification of gelatin degradation assays in HCC cells with or without modulated THBS2 expression. Coomassie blue was used as the loading control. White arrows point to the area of degraded gelation. Scale bar: 10 µm. EV refers to the empty vector, OE refers to THBS2 overexpression, NTC refers to the nontarget control, and 1137 and 2606 refers to the two THBS2 shRNA knockdown clones. Data expressed as the mean ± SEM; ^*^ *p* < 0.05, ^**^ *p* < 0.01, and ^***^ *p* < 0.001 from A–C) Student's *t*‐test or H) one‐way ANOVA with Bonferroni's post‐test.

There is now ample evidence to show that CSCs display a unique ability to resist standard chemotherapy and that many chemotherapeutic drugs lead to enrichment of a functional CSC subpopulation. Our previous work and that of others have shown that CD133+ HCC CSCs confer resistance to cisplatin, doxorubicin, and 5‐fluorouracil (5‐FU) via the preferential expression of AKT/PKB survival pathway proteins.^[^ ^20^, ^21^ ^]^ Treatment of PLC8024, MHCC97L, Huh7, SNU182, and SNU475 HCC cells in vitro (**Figure** **1**A) and MHCC97L xenografts intrahepatically in vivo (Figure 1B) with 5‐FU resulted in a markedly increased CD133 expression, which we and others have recently shown to be an important functional liver CSC marker. Notably, the expression of other well‐known liver CSC markers including EpCAM, CD13, CD44, CD90, OV‐6, K19, and ALDH was not consistently altered following 5‐FU treatment, suggesting that the observed phenomenon may be specific for CD133 liver CSCs (Figure S1, Supporting Information). While many studies have reported on the intrinsically altered mechanisms that mediate this chemoresistance, the link between extracellular matrix stiffness, CSCs, and chemoresistance remains relatively unexplored. Increasing evidence suggests that CSCs activate a set of molecular pathways triggered by the unique mechanical properties of the tumor tissue stroma to maintain stemness. Using a fluorescent‐tagged gelatin degradation assay, we found that sorted CD133+ liver CSCs in both Huh7 and PLC8024 HCC cell line populations displayed an increased ability to degrade the ECM compared to their CD133− non‐CSC counterparts (Figure 1C). Subsequently, to delineate the mechanism by which this process takes place, we performed focused PCR array profiling on 84 targeted genes related to the extracellular matrix and adhesion molecules and identified 8 commonly dysregulated (3 downregulated and 5 upregulated) genes in CD133+ versus CD133− cells isolated from at least 2 of 3 HCC cell lines (Huh7, PLC8024, and SNU182 cells). Of these genes, THBS2 ranked as the “top‐hit” with regard to its significant downregulation that was observed in all 3 cell lines (Figure 1D). THBS2 downregulation in the CD133+ liver CSC subpopulation was validated in the original 3 HCC cell lines used for array profiling, as well as in another HCC cell line, SNU475 cells, at the secretory protein level by Western blot analysis (Figure 1E). Of the 84 genes included in the PCR array, THBS1, which is a close family member of THBS2, was also assessed. Notably, it was not consistently altered in the CD133‐expression HCC subpopulation (Figure 1D, heatmap illustrates all commonly altered genes between any two HCC cell lines and THBS1 is not included). Figure 1F shows an image of the project rationale and how THBS2 was identified for further study.

THBS2 is a member of the thrombospondin family of secreted calcium‐binding glycoproteins that mediate cell–cell and cell–matrix interactions. It is known to function as a potent inhibitor of tumor growth and angiogenesis in a number of cancer types by interacting with MMPs and matrix serine proteases. THBS2 is also widely recognized as an ECM‐modifying enzyme.^[^ ^22^ ^]^ Consistent with these THBS2 roles, we found that lentiviral‐based stable overexpression of THBS2 in HCC cells (Huh7, PLC8024, and MHCC97L) suppressed the activity levels of both MMP9 and MMP2 and led to the degradation of the ECM, as evidenced by gelatin zymography and degradation assays, respectively (Figure 1G,H). Rescue experiments involving lentiviral‐based stable knockdown of THBS2 using two distinct shRNA sequences (1137 and 2606) in THBS2‐overexpressing HCC cells reversed the functional phenotypes (Figure 1G,H). Notably, only MMP2 and MMP9 activity, not their expression, was altered following THBS2 manipulation (Figure S2A, Supporting Information). Knockdown and overexpression of THBS2 resulted in the attenuation and overexpression of THBS2 expression but had no effect on its close family member THBS1, providing evidence that the effects observed are due solely to THBS2 (Figure S2B, Supporting Information).

### 2.2 THBS2 deficiency promotes the cancer and stemness properties of HCC cells grown in 3D matrix gel via ECM remodeling

**Figure 2:**
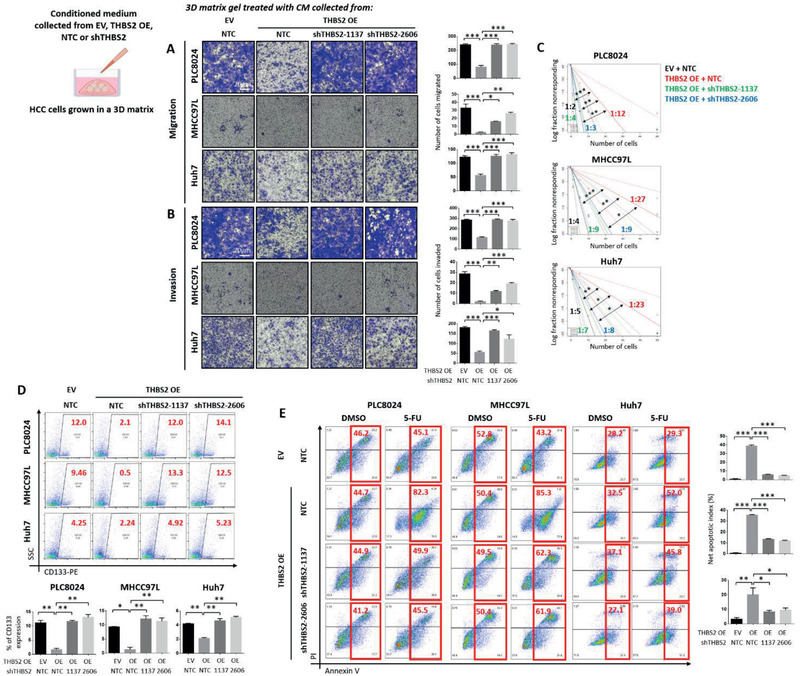
Conditioned medium enriched with THBS2 regulates cancer and stemness properties in HCC cells via matrix gel modification. HCC cells treated with conditioned medium (CM) collected from corresponding HCC cells with THBS2 stably overexpressed or repressed grown in 3D matrix gel. Representative images and quantification of the number of cells that A) migrated or B) invaded. C) In vitro limiting dilution spheroid analysis. D) Representative flow cytometry dot plots and quantification of CD133 expressing cells. E) Representative Annexin V apoptosis dot plots and quantification following treatment with 5‐fluorouracil (5‐FU). EV refers to empty vector, OE refers to THBS2 overexpression, NTC refers to nontarget control, 1137 and 2606 refer to two THBS2 shRNA knockdown clones, PI refers to propidium iodide, and 5‐FU refers 5‐fluorouracil. Data expressed as the mean ± SEM; ^*^ *p* < 0.05, ^**^ *p* < 0.01, and ^***^ *p* < 0.001 from A,B,D,E) one‐way ANOVA with Bonferroni's post‐test or C) chi‐square test.

Next, we explored the ability of THBS2 to regulate functional cancer stemness properties. We carried out in vitro functional tests by treating HCC cells embedded in 3D matrix gel with conditioned medium (CM) collected from HCC cells with or without modulated THBS2, as well as the addition of recombinant THBS2 (rTHBS2). Huh7, PLC8024, and MHCC97L HCC cells cultured in matrix gel and treated with the conditioned medium collected from HCC cells stably overexpressing THBS2 resulted in the diminished ability of the cells to migrate (**Figure** **2**A), invade (Figure 2B), and self‐renew in a limiting dilution spheroid assay (Figure 2C). THBS2 overexpression also resulted in a marked decrease in CD133 expression (Figure 2D) as well as attenuated ability of these cells to resist standard 5‐FU chemotherapy (Figure 2E). Importantly, the rescue of THBS2 knockdown in these THBS2‐overexpressing cells completely reversed the observed functional phenotypes (Figure 2A–E). Notably, overexpression of THBS2 in 5‐FU treatment‐enriched CD133+ HCC cells could sensitize the cells to chemotherapy (Figure S3, Supporting Information), further suggesting the significance of THBS2 in mediating this effect. Consistently, the addition of rTHBS2 to both HCC cells and patient‐derived HCC organoids grown in 3D matrix gel also profoundly reduced the ability of the cells to migrate (Figure S4A, Supporting Information), invade (Figure S4B, Supporting Information), self‐renew (Figure S4C, Supporting Information), and resist 5‐FU treatment (Figure S4D, Supporting Information), and led to a decrease in CD133 expression (Figure S4E, Supporting Information). Of interest, rTHBS2 had no effect on the abovementioned cancer and stemness properties in cells cultured in 2D or in the presence of a RHO/ROCK pathway inhibitor (Y‐27632), suggesting that the ECM modification may represent an important process in THBS2‐mediated change in ability of HCC cells to migrate, invade, self‐renew, and resist 5‐FU treatment (Figure S5, Supporting Information).

### 2.3 THBS2 deficiency promotes collagen degradation and decreases matrix stiffness and metastatic dissemination of HCC tumors

**Figure 3:**
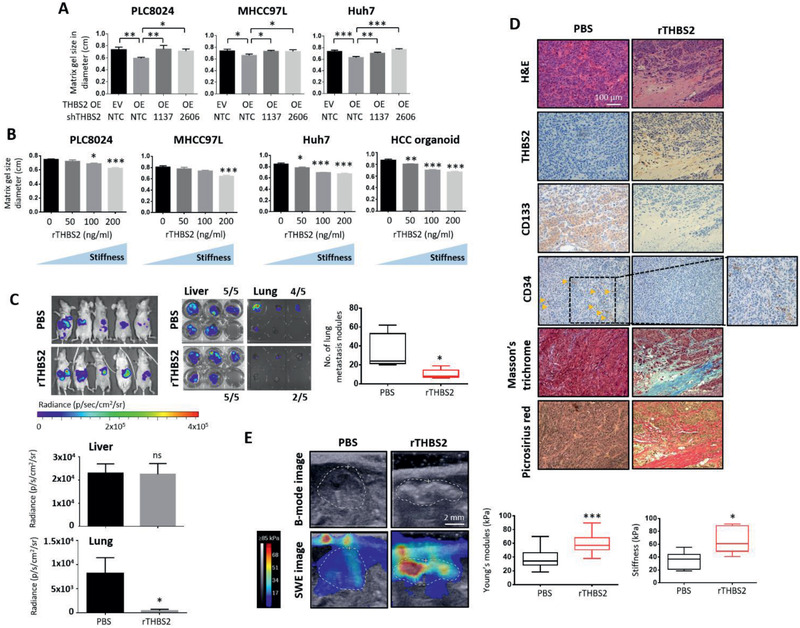
THBS2 deficiency promotes collagen degradation and decreases matrix stiffness and metastatic dissemination of HCC tumors. A) Collagen gel contraction assay of HCC cells with or without THBS2 expression modulation and grown in 3D matrix gel. B) Collagen gel contraction assay of the HCC cells and patient‐derived organoids grown in 3D matrix gel in the absence or presence of varying concentrations of recombinant THBS2 (rTHBS2). C) In vivo and ex vivo images of the luciferase signal in BALB/C nude mice intrahepatically injected with 400 000 MHCC97L HCC cells primed with recombinant THBS2 (rTHBS2)‐modified matrix gel. PBS was used as a control. Box plot on the right showing the number of lung metastasis nodules recorded. Graph plots at the bottom showing the bioluminescence signal of the ex vivo imaged livers and lungs resected. *n* = 5 mice per group. D) Representative images showing H&E, Masson's trichrome and picrosirius red staining, and immunohistochemical staining for THBS2, CD133, and CD34 expression in serial sections of orthotopic liver xenograft tumors. Scale bar: 100 µm. E) Representative elastography images showing the size of tumors (B‐mode image analysis) and stiffness of the tumor tissue (SWE image analysis). Scale bar: 2 mm. The mean elasticity values reflecting the stiffness of the tumors are presented in the box plot (left). Box plots (right) show the stiffness measurements of ex vivo livers harvested from the indicated mouse models as determined by indenter tests. EV refers to empty vector, OE refers to THBS2 overexpression, SWE refers to shear wave elastography, NTC refers to nontarget control, 1137 and 2606 refer to two THBS2 shRNA knockdown clones, and rTHBS2 refers to recombinant THBS2. Data expressed as the mean ± SEM; ^*^ *p* < 0.05, ^**^ *p* < 0.01, and ^***^ *p* < 0.001 from C,E) Student's *t*‐test or A,B) one‐way ANOVA with Bonferroni's post‐test.

Consistent with what has been reported in the literature,^[^ ^23^, ^24^, ^25^ ^]^ we showed that THBS2 can suppress the activity levels of both MMP9 and MMP2 and degrade the ECM (Figure 1G,H). Since the amount of collagen deposition (i.e., connective tissue) is correlated with stiffness, we sought to determine whether modulation of matrix stiffness as a result of ECM remodeling is critical in mediating the effects of THBS2 in HCC. THBS2 overexpression resulted in a significant decrease in matrix gel size (**Figure** **3**A), which was correlated with increased stiffness. Notably, the gel contraction assays, as carried out here, are commonly used by researchers to study ECM remodeling in HCC,^[^ ^26^, ^27^ ^]^ where an increase in gel contraction demonstrates a profibrotic feature.^[^ ^28^ ^]^ Consistently, when HCC patient‐derived 3D organoids^[^ ^29^, ^30^, ^31^ ^]^ or HCC cells were treated with increasing amounts of recombinant THBS2 (0, 50, 100, and 200 ng mL^−1^), a stepwise reduction in matrix gel, which corresponded to enhanced stiffness, was observed (Figure 3B). Our data collectively suggest that altering THBS2 levels in HCC will change collagen degradation, which correlates with cell contractility,^[^ ^32^ ^]^ and influence migration and invasion.

To further demonstrate the link between THBS2, matrix stiffness, and aggressive cancer properties in HCC, we performed an in vivo orthotopic liver metastasis model in which we injected MHCC97L‐expressing HCC cells primed with a recombinant THBS2 (rTHBS2)‐modified matrix gel in vivo directly into the liver. More specifically, for this in vivo animal model setup, MHCC97L cells were first grown in a 3D matrix gel and treated with either a PBS control or rTHBS2. These PBS or rTHBS2 “primed” cells were then injected intrahepatically into immunodeficient mice. rTHBS2 did not alter the ability of these cells to form tumors as evidenced by similar bioluminescence signal (Figure 3C, liver bar graph at the bottom), but greatly reduced the capacity of the cells to metastasize to the lung (Figure 3C, box plot on the right and lung bar graph at the bottom). Histological analysis of the resected tumors showed a decreased microvessel density count and increased connective tissue (Figure 3D). HCC livers in mice injected with rTHBS2 primed cells displayed stiffer tumors than HCC livers in mice injected with PBS control cells (Figure 3E; Figure S6B, Supporting Information). Most interestingly, we were able to consistently observe that CD133 expression was reduced in tumors formed by HCC cells overexpressing THBS2 or primed with rTHBS2 (Figure 3D; Figure S7B, Supporting Information). Similar results were also obtained when we injected luciferase‐labeled MHCC97L HCC cells that stably overexpressed empty vector (EV) control or THBS2 intrahepatically. THBS2 overexpression did not alter the ability of these cells to form tumors in the liver (5 of 6 in the EV vs 6 of 7 in the THBS2 OE group) but significantly attenuated the ability of these cells to metastasize to the lung (5 of 6 in the EV vs 2 of 7 in the THBS2 OE group), as evidenced by both ex vivo bioluminescence and histological examination of the number of lung metastatic nodules on resected xenograft tumors (Figure S7A, Supporting Information). Immunohistochemical analysis of the resected liver tumors revealed a marked decrease in microvessel density count, as demonstrated by CD34 level and an increase in connective tissue, as demonstrated by Masson's trichrome and picrosirius red staining (Figures S6A and S7B, Supporting Information). We subjected the liver tumors to shear wave elastography imaging and indenter test assays for stiffness measurements. Tumors overexpressing THBS2 were significantly stiffer than tumors formed by the injected EV control cells, further confirming that the level of collagen fiber deposits is positively correlated with tumor stiffness (Figure S7C, Supporting Information).

### 2.4 Recombinant THBS2 sensitizes HCC tumors to 5‐FU chemotherapy, diminishes their metastatic ability, and reduces CD133 expression in vivo

**Figure 4:**
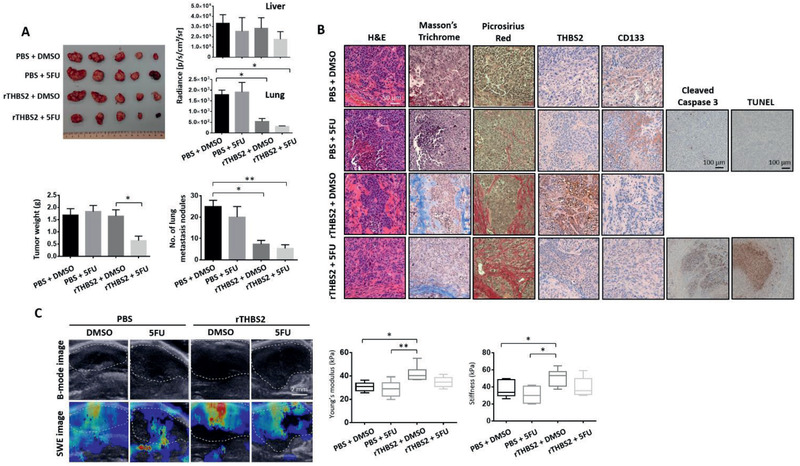
Recombinant THBS2 sensitizes HCC tumors to 5‐FU chemotherapy, diminishes their metastatic ability, and reduces CD133 expression in vivo. A) Images of resected liver tumors of BALB/C nude mice intrahepatically injected with 400 000 MHCC97L cells primed with or without recombinant THBS2 (rTHBS2)‐modified matrix gel and with or without 5‐FU treatment. Graph plots of the bioluminescence signal of the ex vivo imaged livers and lungs resected from BALB/C nude mice intrahepatically injected with MHCC97L cells primed with or without recombinant THBS2 (rTHBS2)‐modified matrix gel and with or without 5‐FU treatment. PBS/DMSO was used as a control. Box plots showing the tumor weight in the liver and the number of lung metastasis nodules. *n* = 5 mice per group. B) Representative images showing H&E, Masson's trichrome and picrosirius red staining, and immunohistochemical staining for THBS2, CD133, cleaved caspase 3 expression, as well as apoptosis by TUNEL as in serial sections of orthotopic liver xenograft tumors. Scale bar: 50 µm, with the exception of cleaved caspase 3 and TUNEL where scale bar is at 100 µm. C) Representative elastography images showing the size of the tumors (B‐mode image analysis) and the stiffness of the tumor tissue (SWE image analysis). Scale bar: 2 mm. The mean elasticity values reflecting the stiffness of the tumors are presented in the box plot (left). Box plots (right) show the stiffness measurements of ex vivo livers harvested from the above mouse model by indenter tests. SWE refers to shear wave elastography, rTHBS2 refers to recombinant THBS2, and 5‐FU refers to 5‐fluorouracil. Data expressed as the mean ± SEM; ^*^ *p* < 0.05, ^**^ *p* < 0.01, and ^***^ *p* < 0.001 from one‐way ANOVA with Bonferroni's post‐test.

We then questioned whether rTHBS2, when used in combination with 5‐FU, can sensitize HCC tumors to chemotherapy, eradicate the CSC subpopulation that we found had been enriched due to 5‐FU treatment alone, and/or decrease metastasis. Indeed, treating HCC cells and organoids with a combination of rTHBS2 and 5‐FU resulted in a marked decrease in CD133 expression, as evidenced by an in vitro 3D matrix gel culture setup (Figure S4D, Supporting Information) and an in vivo intrahepatic HCC animal model. For the in vivo animal model setup, MHCC97L cells were first grown in a 3D matrix gel and treated with either PBS control or rTHBS2. These PBS or rTHBS2 “primed” cells were then injected intrahepatically into immunodeficient mice, which were subsequently treated with 5‐FU following tumor detection. The rTHBS2 and 5‐FU combination treatment resulted in a marked reduction not only in tumor size and the number of lung metastatic nodules (**Figure** **4**A) but also in CD133 expression, while enhancing collagen fiber deposition, as shown by Masson's trichrome staining (Figure 4B; Figure S6C, Supporting Information). Cleaved caspase 3 immunostaining and TUNEL assays showed increased apoptosis in the rTHBS2+5‐FU treatment group as compared to PBS+5‐FU treatment group (Figure 4B). Interestingly, only the rTHBS2‐treated cells formed collagen fiber deposit‐enriched HCC tumors, and the tumors that were generated by the PBS‐treated control cells showed no collagen formation. rTHBS2 treated cells also formed stiffer tumors, as evidenced by elastography imaging and indenter test assays (Figure 4C). 5‐FU treatment of the mice injected with PBS‐primed HCC cells resulted in an enrichment of CD133 expressing cells (Figure 4B), which is consistent with our previous findings showing the CD133+ liver CSCs conferred chemoresistance.^[^ ^20^, ^21^ ^]^ Our results revealed that rTHBS2 sensitized and enhanced the efficacy of the 5‐FU treatment and reduced CD133 expression, which we now know represents the root of the disease. Notably, we observed more collagen deposition in both rTHBS2‐treated groups, as evidenced by the increased picrosirius red staining; however, only the rTHBS2+DMSO group showed increased stiffness, as measured by the elastography and indenter assays. We rationalize that the measurements provided by the elastography and indenter assays represent collective measurements of the entire tumor mass, whereas picrosirius red and trichrome staining reveal the thickness of the collagen fibers inside the tumor. For all the rTHBS2 group‐treated mice, thick collagen fibers likely developed in the tumors as a result of the recombinant THBS2 treatment prior to the chemotherapy treatment. Chemotherapy treatment induces apoptosis without affecting collagen thickness. However, a past study did find chemotherapy induced apoptosis in cancer cells to result in decreased stiffness,^[^ ^33^ ^]^ which may explain for the high collagen deposition detected in both the rTHBS2+DMSO and rTHBS2+5‐FU groups as confirmed by picrosirius red staining, yet only the rTHBS2+DMSO group showed tumors with increased stiffness.

### 2.5 THBS2 expression is frequently downregulated in HCC, and its deficiency is tightly associated with low HCC survival

**Figure 5:**
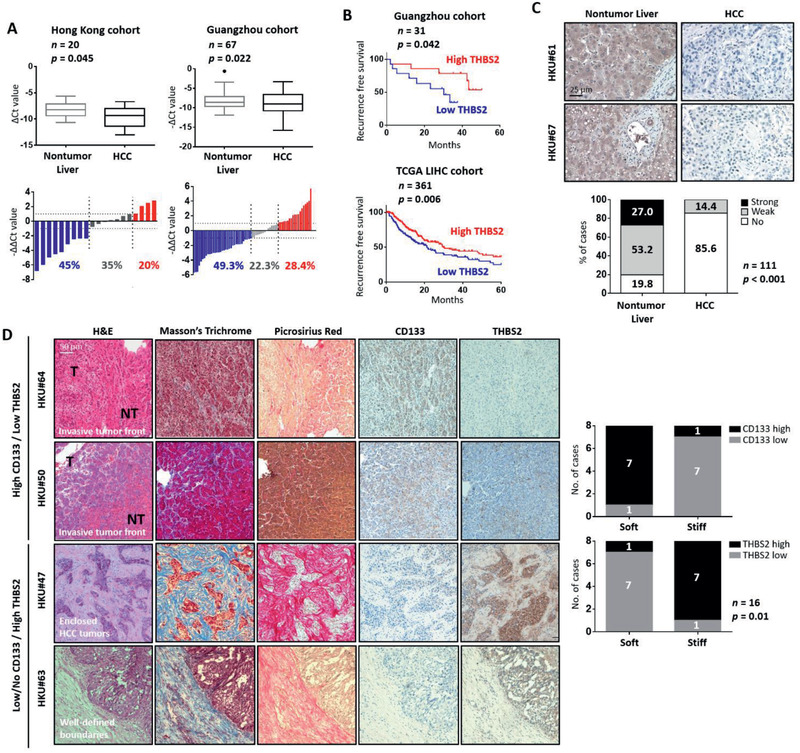
THBS2 expression is frequently downregulated in HCC, and its deficiency is tightly associated with low HCC survival. A) Box and waterfall plot analyses of THBS2 mRNA expression in nontumor liver and matched HCC tissue from patients in both the Hong Kong (*n* = 20) and Guangzhou (*n* = 67) cohorts. B) Kaplan–Meier survival analysis comparing recurrence‐free survival of HCC patients with different THBS2 mRNA expression levels in the Hong Kong (*n* = 31) and TCGA LIHC (liver ‐ hepatocellular carcinoma) (*n* = 361) cohorts. C) Representative immunohistochemical analysis of THBS2 expression in HCC tumor and adjacent nontumor liver tissue. Scale bar: 25 µm. Box plot showing the distribution of THBS2 expression levels at different intensities in the tissue microarray of 111 matched nontumor liver and HCC clinical samples. D) Histological examination of areas in HCC clinical tissue samples with high CD133 expression and low THBS2 expression (top) or absent/low CD133 expression and high THBS2 expression (bottom). The samples were stained with Masson's trichrome and picrosirius red stains for visualization of the connective tissue and collagen fiber deposits as well as for CD133 and THBS2. Scale bar: 50 µm. Box plot showing the number of cases showing high and low CD133 expression or high and low THBS2 expression in soft or stiff HCC tumors (*n* = 8 matched cases examined). Data expressed as the mean ± SEM; ^*^ *p* < 0.05, ^**^ *p* < 0.01, and ^***^ *p* < 0.001 from Student's *t*‐test.

To investigate the clinical significance of THBS2, we used qPCR to examine THBS2 mRNA expression in primary HCC and matched nontumor liver tissues from clinical samples collected from both Hong Kong and Guangzhou. THBS2 downregulation of ≥2‐fold was displayed in >45% of the HCC specimens compared with nontumor specimens in both cohorts (**Figure** **5**A). Analysis of data obtained in‐house and from a publicly available dataset (The Cancer Genome Atlas (TCGA), Liver Cancer (LIHC)) also showed THBS2 downregulation in HCC to be correlated with the worst recurrence‐free survival (Figure 5B). These observations were confirmed by immunohistochemistry in a separate cohort consisting of 111 primary HCC and adjacent nontumor tissues, in which significantly more HCC cases were found to express no (85.6%) or weak (14.4%) THBS2 compared to the matched nontumor liver samples (19.8% no, 53.2% weak, and 27% strong) (Figure 5C; Figure S8, Supporting Information). Upon in‐depth analysis of the clinical samples, we found that areas in HCC samples that displayed high CD133 and low THBS2 expression had softer tumors (less collagen fiber deposition) and fewer confined tumors with a clear invasive tumor front showing the direction by which HCC cells migrate from the tumor to the adjacent nontumor areas (Figure 5D top; Figure S6D, Supporting Information). In contrast, areas in HCC samples that display absent or low CD133 and high THBS2 expression showed extensive collagen fiber formation and thus stiffer tumors, with the HCC cells confined in lobules without an aggressive invasive front (Figure 5D bottom; Figure S6D, Supporting Information). A soft spot in an HCC tumor was also significantly correlated with high CD133 and low THBS2 expression (Figure 5D graphs on the right; *n* = 16; *p* = 0.01).

### 2.6 A soft microenvironment promotes aggressive cancer properties, induces CD133 expression, and suppresses THBS2 expression through histone H3 modifications

**Figure 6:**
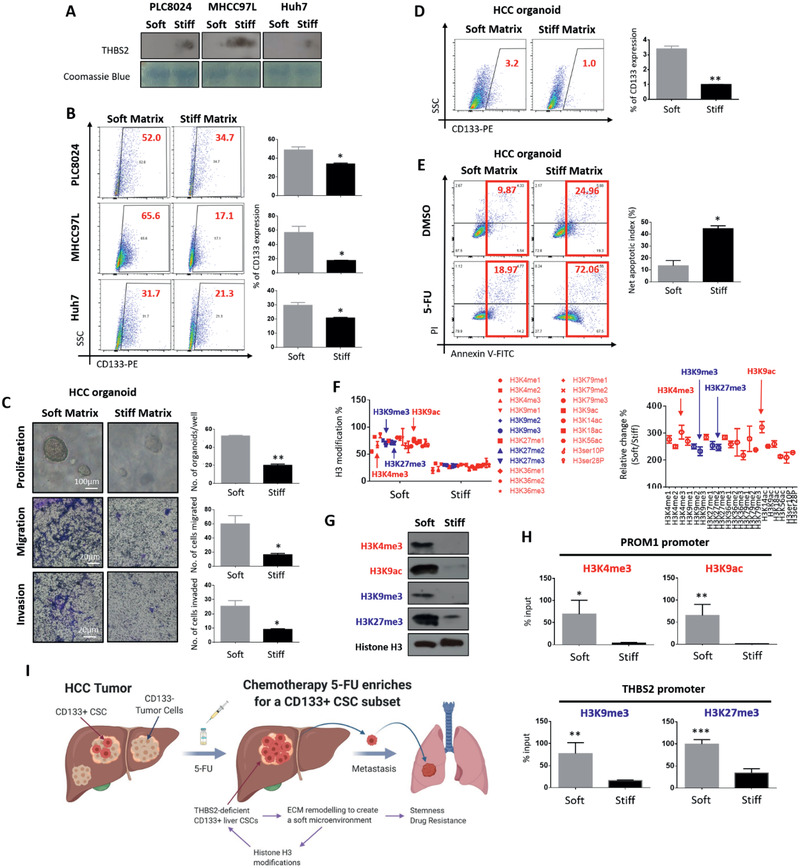
A soft microenvironment promotes aggressive cancer properties, induces CD133 expression, and suppresses THBS2 expression through histone H3 modifications. A) Western blots showing the secreted THBS2 proteomic expression in HCC cells grown in soft (0.25 kPa) or stiff (15 kPa) matrix. Coomassie blue was used as the loading control. B) Representative flow cytometry dot plots and quantification of CD133 expression in HCC cells grown in soft and stiff matrix. C) Representative images and quantification of the patient‐derived HCC organoids grown in soft (0.25 kPa) or stiff (15 kPa) matrix. Images show organoid size. The bar graph shows the number of organoids recorded per 1000 cells. Representative images and quantification of the number of patient‐derived HCC organoids that migrated and invaded when grown in soft or stiff matrix. Scale bar: 100 µm for proliferation and 20 µm for migration and invasion. D) Representative flow cytometry dot plots and quantification of CD133 expression in the patient‐derived HCC organoids grown in soft or stiff matrix. E) Representative Annexin V apoptosis dot plots and quantification of patient‐derived HCC organoids grown in soft or stiff matrix following treatment with 5‐fluorouracil (5‐FU). 5‐FU refers to 5‐fluorouracil and PI refers to propidium iodide. F) Graph plots of absolute H3 modification and relative changes to H3 modifications in MHCC97L cells cultured in soft or stiff matrix. G) Western blot validation of global changes in transcriptional activation (H3K4me3 and H3K9ac) and transcriptional silencing marks (H3K9me3 and H3K27me3) in MHCC97L cells cultured in soft or stiff matrix. H) Bar graph summary of ChIP‐qPCR analysis of the enrichment of transcriptional activation (H3K4me3 and H3K9ac) and transcriptional silencing marks (H3K9me3 and H3K27me3) at the respective promoter regions of PROM1/CD133 and THBS2, respectively. I) Schematic diagram illustrating the findings of this study showing that extracellular matrix remodeling by chemotherapy 5‐FU‐enriched THBS2‐deficient CD133 cancer stem cells can provide a route of escape that leads to HCC metastasis. In brief, 5‐FU enriches for CD133 expressing cells with deficient THBS2 expression. THBS2, which is a known extracellular matrix (ECM)‐modifying protein, has the ability to alter MMP2/MMP9 activity and thus collagen and matrix stiffness, which leads to altered cancer and stemness properties. This local soft microenvironment then induces mechanoepigenetic changes to enhance CD133 expression and suppress THBS2 expression through histone H3 modifications at their respective promoter regions, facilitating a positive feedback loop that supports the clonogenic expansion of a subpopulation of HCC cells with high CD133 expression and low THBS2 expression. Data expressed as the mean ± SEM; ^*^ *p* < 0.05, ^**^ *p* < 0.01, and ^***^ *p* < 0.001 from Student's *t*‐test.

We first cultured HCC cells on mechanically tunable 3D collagen in a Matrigel setting and found softer support (0.25 kPa) to promote CD133 expression, concomitant with decreased THBS2 secretion, compared to stiffer support (15 kPa)^[^ ^34^ ^]^ (**Figure** **6**A,B). We also cultured patient‐derived HCC organoids on this same setup and found softer support to promote HCC cell proliferation, migration, invasion, CD133 expression, and the ability to resist 5‐FU chemotherapy compared to stiffer support (Figure 6C–E). Recent studies have shown that a soft matrix promotes histone modifications to induce the expression of the stemness‐related gene Sox2 in melanoma cells^[^ ^5^ ^]^ and pluripotency‐related genes in mesenchymal stem cells.^[^ ^35^ ^]^ To explore the mechanism by which a soft matrix controls the CD133 and/or THBS2 expression, we investigated the differences in H3 modifications between HCC cells cultured on soft and stiff matrixes using a histone H3 modification multiplex assay kit that detects and quantifies 21 modified histone H3 patterns (Figure 6F). Interestingly, all the tested H3 modifications were drastically increased when HCC cells were cultured in a soft matrix gel. We then examined publicly available ChIP‐seq data of HepG2 HCC cells and data extracted from ENCODE histone modification tracks by the Broad Institute using Integrative Genomics Viewer software. Among all the H3 modification markers available, the transcriptional activation marks H3K4me3 and H3K9ac were identified in the PROM1/CD133 promoter region, while transcriptional silencing marks H3K9me3 and H3K27me3 were found in the THBS2 promoter region (Figure 6H; Figure S9, Supporting Information). Subsequent Western blot analysis confirmed the global upregulation of these 4 marks in HCC cells cultured in soft matrix (Figure 6G). ChIP‐qPCR results confirmed that the PROM1/CD133 promoter is enriched with the transcriptional activation mark H3K4me3 and H3K9ac, while THBS2 is enriched with the transcriptional silencing marks H3K9me3 and H3K27me3 (Figure 6H). These data suggest that matrix softness induces mechanoepigenetic changes that enhance CD133 expression and suppress THBS2 expression through histone H3 modifications at their respective promoter regions. Collectively, our work demonstrates that a soft environment induces cancer stem cell‐like characteristics and promotes the ability of HCC cells to disseminate and metastasize. These effects are mediated by altered MMP activity and collagen/ECM degradation of a CD133+ CSC subpopulation that are deficient in THBS2 in HCC tumors. This local soft environment then induces mechanoepigenetic changes that enhance CD133 expression and suppress THBS2 expression through histone H3 modifications at their respective promoter regions, facilitating a positive feedback loop to support clonogenic expansion of a subpopulation of HCC cells with high CD133 expression and low THBS2 expression (Figure 6I).

## 3 Conclusions

Matrix stiffness is a vital physical microenvironment in solid cancer. Yet, its impact on cancer stemness remains controversial. In the context of HCC, a disease that is uniquely linked to matrix stiffness because of the underlying prevalence of liver cirrhosis/fibrosis in the majority of HCC patients, a handful of studies have reported controversial findings linking either matrix stiffness or softness to cancer stemness. In support of the widely accepted belief that liver stiffness measurement is a strong predictor of HCC development, a number of studies have found that a stiffer matrix upregulates vascular endothelial growth factor expression in HCC cells,^[^ ^36^ ^]^ promotes latent TGF‐*β*1 activation to support liver cancer development,^[^ ^37^ ^]^ and enhances the proliferation, migration^[^ ^38^ ^]^ and sorafenib resistance of HCC cells.^[^ ^39^ ^]^ Using a 2D in vitro culture system with tunable stiffness, You et al. also found that matrix stiffness promotes stemness characteristics of HCC cells by enhancing CD133/EpCAM expression in CSCs and functional stemness properties by driving integrin *β*1/AKT/mTOR/SOX2 signaling.^[^ ^17^ ^]^ However, in direct contrast to these reports, a number of studies have found that a softer matrix support enhances the number of HCC cells with a CSC phenotype.^[^ ^18^, ^19^ ^]^ As aforementioned, these studies have primarily been limited to 2D culture experimental setups in vitro using either polyacrylamide gel or hydrogel coated with collagen I or polyacrylamide gel without collagen I. The 2D cell culture model has been criticized for being nonphysiological due to the lack of 3D architecture. More advanced studies have used a soft 3D gel to promote cancer properties and upregulate stem cell markers (i.e., CD133, nestin, c‐kit, and BMI‐1) in melanoma cells,^[^ ^40^ ^]^ suggesting that a 3D soft environment promotes CD133‐expressing tumor‐repopulating cells.

In light of the controversy in the field and because of improved culture setups and in vivo measurements, this study adopted a 3D tunable matrix gel system previously utilized by Grasset et al., which includes type I collagen to better mimic the liver microenvironment.^[^ ^34^ ^]^ This system also fit well into the HCC patient‐derived organoid culture setup recently established in our laboratory in collaboration with Hans Clevers,^[^ ^31^ ^]^ which was shown to provide 3D architecture similar to in vivo structures. Furthermore, our present study is also one of the few that has adopted in vivo elastography stiffness measurements in mouse liver. Our current study reveals a critical role for the chemotherapy‐enriched CD133 expressing cells that preferentially secrete less ECM‐modifying enzyme THBS2 to promote aggressive cancer and stemness properties in HCC. The CSC subpopulation remodels the ECM by changing MMP activity, collagen degradation, and matrix stiffness. The local soft spot created by liver CSCs can enhance stemness and drug resistance and provides a route of cell escape to facilitate HCC metastasis to the lung. Interestingly, we identified a positive feed‐forward loop where a local soft spot microenvironment in the HCC tumor will, through mechanoepigenetics, in particular histone H3 modifications, lead to an enrichment of CD133 expressing cells that secrete markedly less THBS2 for ECM modification, allowing the maintenance of a local matrix soft spot. It will be interesting to further explore the exact mechanism by which secreted THBS2 suppresses expression of MMP2/9 and CD133 or how CD133 inhibits THBS2 expression in HCC cells in the future. Of interest, THBS2 has been suggested to alter MMP2 by binding to it and directing it to scavenger receptors like LRP on cell surfaces, resulting in its clearance from the ECM.^[^ ^25^ ^]^ In regard to regulation of THBS2, recent studies have shown THBS2 to be regulated by a number of microRNAs including miR‐744‐5p,^[^ ^41^ ^]^ miR‐1246,^[^ ^42^ ^]^ miR‐221‐3p,^[^ ^43^ ^]^ and miR‐20a.^[^ ^44^ ^]^ Of note, our group has previously reported CD133+ liver CSCs to preferentially express miR‐1246 in HCC;^[^ ^45^ ^]^ but whether miR‐1246 targets THBS2 in HCC will need to be further explored. When HCC cells and patient‐derived organoids are cultured on 2D plastic or 3D matrix gel but in the presence of a RHO/ROCK inhibitor, the functional alterations were not observed, suggesting that ECM modification may be required for THBS2‐mediated change in ability of HCC cells to migrate, invade, self‐renew, and resist 5‐FU treatment. It should however be noted that Y‐27632, the ROCK inhibitor that was used in this study, exhibits off‐target effects, targeting protein kinases like PKA,^[^ ^46^, ^47^, ^48^ ^]^ PKC,^[^ ^47^ ^]^ as well as PRK‐2.^[^ ^49^ ^]^ As such, the actual involvement of Rho/ROCK signaling in THBS2‐mediated cancer and stemness phenotype will need to be further supported by additional specific Rho/ROCK assays in the future.

We also demonstrated that THBS2 downregulation in HCC is tightly associated with adverse HCC survival and that high CD133/low THBS2 expression in HCC specimens correlates with decreased collagen fiber deposits and invasive tumor fronts, which supports our functional findings in vitro and in vivo. Our findings suggest that the distribution of CD133+ liver CSCs in HCC tissue is not uniform, is likely maintained in a local soft environment and is distributed at the invasive tumor front. Our findings also have implications for the treatment of cancer stemness and for the prevention of tumor outgrowth by disseminated tumor cells. As a proof‐of‐concept, we have demonstrated that rTHBS2 is effective in sensitizing HCC tumors to 5‐FU chemotherapy and in diminishing CD133 expression concomitant with enhanced tissue stiffness. Thus, it may be therapeutically appropriate to consider ways to enhance THBS2 expression in HCC and use it in combination with 5‐FU. In 2017, Liao et al. reported on a methodology to activate endogenous target genes through transepigenetic remodeling in vivo. The system is based on the recruitment of Cas9 and transcriptional activation complexes to target loci by modified single‐guide RNAs. The authors successfully applied the technique to treat mouse models of diabetes, acute kidney disease, and muscular dystrophy as a proof‐of‐concept.^[^ ^50^ ^]^ This tool can be considered for use for THBS2 activation in vivo for HCC patients and as a combination treatment along with the use of 5‐FU for anticancer stemness targeting in HCC. Notably, we did attempt to examine whether our findings are also relevant to other chemotherapy drugs, such as cisplatin, or molecular targeted drugs, such as sorafenib; but it seems that our observations are 5‐FU‐specific.

THBS2 is a member of the thrombospondin family of multidomain and secreted multicellular calcium‐binding glycoproteins that mediate cell‐to‐cell and cell‐to‐matrix interactions. It functions as a potent inhibitor of tumor growth and angiogenesis in a number of cancers, by interacting with MMPs and matrix serine proteases such as plasminogen activator and is recognized as an important ECM‐modifying enzyme.^[^ ^51^, ^52^ ^]^ Although the association between THBS2 and ECM modification has been established, the link between THBS2 and matrix stiffness/softness has never been explored and remains a point of novelty in our current work. In summary, our study clarifies the anonymity between matrix stiffness and HCC stemness and offers a new regulatory pathway to direct HCC stemness.

## 4 Experimental Section

##### Reagents

5‐FU was purchased from Sigma‐Aldrich (Cat No. F6627). Human recombinant thrombospondin‐2 (rTHBS2) was purchased from R&D Systems (Cat No. 1635‐T2). Y27632 was purchased from AbMole (Cat No. M1817).

##### Cell Lines and HCC Patient‐Derived Organoid Cultures

The PLC8024 human HCC cell line was obtained from the Institute of Virology, Chinese Academy of Medical Sciences, Beijing, China. The Huh7 HCC cell line was provided by H. Nakabayashi, Hokkaido University School of Medicine, Japan. The MHCC97L HCC cell line was obtained from the Liver Cancer Institute, Fudan University, China. The SNU182 and SNU475 HCC cell lines were purchased from American Type Culture Collection (ATCC). In addition, 293T and 293T/17 cells were purchased from the ATCC, while 293FT cells were purchased from Invitrogen. The cell lines used in this study were regularly authenticated by morphological observation and tested for the absence of mycoplasma contamination. For patient‐derived organoid cultures, the cells were isolated and cultured as previously described.^[^ ^29^, ^30^, ^53^ ^]^ HCC tissues used for organoid establishment were obtained from patients undergoing hepatectomy or liver transplantation at Queen Mary Hospital, Hong Kong, with informed consent obtained from all patients and protocols approved by the Institutional Review Board of the University of Hong Kong/Hospital Authority Hong Kong West Cluster (Ethics ID: UW 05‐359 T/1022). The samples were collected from patients who had not received any previous local or systemic treatment prior to surgery.

##### Patient Samples

For the qPCR analysis, primary human HCC and adjacent nontumor liver tissue samples were obtained from 87 patients undergoing surgical resection at either the Queen Mary Hospital in Hong Kong or the Sun Yat‐Sen University Cancer Centre in Guangzhou, China. For immunohistochemistry analysis, human primary human HCC and adjacent nontumor tissue samples were obtained from patients undergoing hepatectomy at either the Queen Mary Hospital in Hong Kong or the Sun Yat‐Sen University Cancer Centre in Guangzhou, China. Informed consent was obtained from all patients before the collection of liver specimens. Specimen collection and all experiments were approved by the Institutional Review Board of the University of Hong Kong/Hospital Authority Hong Kong West Cluster (Ethics ID: UW 05‐359 T/1022) or Sun Yat‐Sen University (Ethics ID: approval letter only). Samples were collected from patients who had not received any previous local or systemic treatment prior to surgery.

##### Animal Models, B‐Mode Ultrasound, and Shear Wave Elastography

The study protocol was approved by and performed in accordance with the Committee on the Use of Live Animals in Teaching and Research at The University of Hong Kong. Metastasis was assessed by orthotopic injection into the liver of 6 week old BALB/c nude mice to observe lung metastasis. Specifically, 400 000 MHCC97L luciferase‐labeled cells expressing EV control or THBS2 were injected into the left lobes of the livers of BALB/C nude mice. Four and eight weeks after implantation, the mice were administered 100 mg kg^−1^ d‐luciferin via peritoneal injection for bioluminescent imaging (IVIS 100 Imaging System, Xenogen) to monitor tumor growth and metastasis. Tumor stiffness was assessed by B‐mode ultrasound and shear wave elastography imaging using an Aixplorer Scanner (SuperSonic Imagine) incorporating a SuperLinear 22‐7lab linear transducer dedicated to small animal imaging. Measurements were taken at 4 and 8 weeks postinjection. Five images were taken for each mouse. The tumor volume was determined by B‐mode ultrasound. Mean elasticity values from the region of interest were collected. Livers and lungs were harvested for ex vivo imaging and histological analysis 8 weeks postinjection.

##### Indenter Test

Exterior stiffness of the tumor was measured using a cylindrical plane‐ended indenter on the mouse skin surface. The tissue was indented at a speed of 0.5 mm s^−1^ for three cycles of loading and unloading with a maximum indentation depth of ≈4 mm. The interior stiffness of the tumor was measured on the harvested orthotopic liver tumor tissue. The tissue was compressed at a speed of 0.5 mm s^−1^ with a maximum force of 0.1 N for three cycles of loading and unloading. The mechanical properties of the tested sample were calculated as the mean of the three tests.

##### Histone H3 Modification Assay

MHCC97L cells were cultured in soft and stiff matrix gels for 7 days. Histones were extracted using an EpiQuik Total Histone Extraction Kit (EpiGentek). Global histone H3 modification in the soft matrix environment was determined using an EpiQuik Histone H3 Modification Multiplex Assay Kit (EpiGentek). The results were read on a VICTOR multilabel plate reader (Perkin Elmer) at 450 nm and normalized based on the total H3 levels.

##### Statistical Analysis

All statistical analyses were performed using GraphPad Prism 6 (GraphPad Software Inc.). All data were expressed as the means ± SEM from at least three independent experiments. Unpaired Student′s *t*‐test or one‐way ANOVA with Bonferroni's post‐test were carried out to compare the differences between two groups. Survival curves were calculated using Kaplan–Meier method, and *p*‐value was determined by log‐rank test. Recurrence‐free survival was calculated from the date of HCC resection to the time of first recurrence or death. Patients who were lost to follow‐up were treated as censored events. Limiting dilution analysis (http://bioinf.wehi.edu.au/software/elda/) was calculated using chi‐square test for pairwise differences in active cell frequency between groups. A *p*‐value of less than 0.05 was considered as statistically significant, and all statistical tests were two‐tailed.

^*^ *p* < 0.05, ^**^ *p* < 0.01, and ^***^ *p* < 0.001. The number of animals included per group can be found in each respective figure.

## Conflict of Interest

The authors declare no conflict of interest.

## Author Contributions

K.‐Y.N. and S.M. conceived the project and designed the studies. K.‐Y.N. performed the research, and analyzed and interpreted the data with the help of T.‐L.W., S.T.L., and M.T. Q.T.S. and Y.‐P.Z. performed the research, and analyzed and interpreted the data relating to the B‐mode ultrasound, shear wave elastography, and indenter test measurements. C.‐M.L., K.M., J.‐P.Y., and X.‐Y.G. obtained patient consent and provided the clinical samples for clinical analysis and organoid culture. T.K.L. provided critical scientific input. K.‐Y.N. and S.M. wrote the paper. S.M. supervised the project and provided funding for the study.

## Supporting information

Supporting Information
