# Supplementary material for: Chemotherapy‐Enriched THBS2‐Deficient Cancer Stem Cells Drive Hepatocarcinogenesis through Matrix Softness Induced Histone H3 Modifications
Source: Adv Sci (Weinh). 2021 Jan 4;8(5):2002483. doi: 10.1002/advs.202002483 (PMC7927606; doi:10.1002/advs.202002483)

© 2020 Wiley-VCH GmbH

Supporting Information

for *Adv. Sci.*, DOI: 10.1002/advs.202002483

Chemotherapy enriched THBS2-deficient cancer stem cells drive hepatocarcinogenesis through matrix softness induced histone H3 modifications

Kai-Yu NG, Queenie T SHEA, Tin-Lok WONG, Steve T LUK, Man TONG, Chung-Mau LO, Kwan MAN, Jing-Ping YUN, Xin-Yuan GUAN, Terence K LEE, Yong-Ping ZHENG, and Stephanie MA[*]

SUPPLEMENTARY INFORMATION

Chemotherapy enriched THBS2-deficient cancer stem cells drive hepatocarcinogenesis through matrix softness induced histone H3 modifications

Kai-Yu NG, Queenie T SHEA, Tin-Lok WONG, Steve T LUK, Man TONG, Chung-Mau LO, Kwan MAN, Jing-Ping YUN, Xin-Yuan GUAN, Terence K LEE, Yong-Ping ZHENG, Stephanie MA

SUPPLEMENTARY EXPERIMENTAL PROCEDURES

Preparation of polyacrylamide gel supports. Polyacrylamide gel was prepared according to a previous publication [54]. Briefly, different ratios of acrylamide and bis-acrylamide (Sigma) were mixed with 10% APS and 1% TEMED in 10 mM HEPES and left for 1 hr at room temperature to set into a gel. The gel was subsequently coated with a Sulfo-SANPAH photoactive cross-linker (Life Technologies) and activated by UV illumination for 10 min, followed by collagen coating. HCC cells in DMEM were seeded into the gels for culturing.

Matrix gel setup and gel contraction/retraction assay. Matrix gel was prepared according to a previous publication [34]. Stiff matrix gel conditions consisted of 4 mg/ml collagen (Corning) and 2 mg/ml Matrigel (Corning), while soft conditions consisted of 1 mg/ml collagen and 0.5 mg/ml Matrigel. HCC cell lines and organoids were embedded in 50 µl of matrix gel in 24-well plates. THBS2 modified matrix gel was prepared by adding rTHBS2 or conditioned medium collected from THBS2 overexpressing cells to matrix gel composed of 4mg/ml collagen and 2 mg/ml Matrigel. For gel contraction/retraction assay, cells were embedded in matrix gel composed of 4 mg/ml collagen and 2 mg/ml Matrigel for 7 days. Gel image was captured with a Gel Doc Image System with Universal Hood II using trans-white illumination (Bio-Rad). The diameter of the gel was measured using ImageJ software.

PCR array profiling. cDNA from three pairs of sorted CD133+HCC cell lines was analyzed for 84 genes important for cell-cell and cell-matrix interactions using the Extracellular Matrix and Adhesion Molecules RT^2 Profiler PCR Array (PAHS-013Z; Qiagen). Briefly, cDNA template was combined with RT^2 SYBR Green qPCR Master Mix (Qiagen) and water. Equal aliquots of this mixture (25 µl) was added to each well of the PCR array plate loaded with gene-specific primer sets. Amplification and detection were performed using an ABI 7900HT Fast Real-Time PCR System. Web-based PCR array data analysis software (www.SABiosciences.com/pcrarraydataanalysis.php) was used for ΔΔCT–based fold-change calculations. Expression values were normalized over the expression of 5 housekeeping genes (ACTB, B2M, GAPDH, HPRT1 and RRLP0) and presented as the fold regulation ratio of CD133+ populations to CD133- populations. For all fold change values greater than 1, the fold regulation and fold change values are the same. For all fold-change values (x) less than 1, the fold regulation is the negative inverse of the fold change ($-1/x$).

The Cancer Genome Atlas (TCGA) data. Gene expression profiles of HCC (*n* = 361) tissue samples were analyzed for the expression of THBS2 transcripts available in the Liver Hepatocellular Carcinoma (LIHC) dataset of the TCGA Research Network database.

Quantitative real-time PCR. Total RNA was extracted using RNA-IsoPlus (TaKaRa), and cDNA was synthesized by PrimeScript RT Master Mix (TaKaRa). qPCR was performed with EvaGreen qPCR Master Mix (ABM) and the following primers: THBS2: (forward 5'- ATAGACAGCTTCGCTCTGGAC -3' and reverse 5'-CAAACCCCTGAAGTGACTCTC-3'); MMP2: (forward 5'- TGGCGATGGATACCCCTTT -3' and reverse 5'-TTCTCCCAAGGTCCATAGCTCAT -3'); MMP9: (forward 5'- AGACCTGGGCAGATTCCAAAC -3' and reverse 5'-CGGCAAGTCTTCCGAGTAGT -3'); and β-ACTIN: (forward 5'- CATCCACGAAACTACCTTCAACTCC -3' and reverse 5'- GAGCCGCCGATCCACACG -3') on a LightCycler 480 II System (Roche) with data analyzed using the LightCycler 480 II software (Roche). Relative expression differences were calculated using the $2^{-\Delta\Delta Ct}$ method.

Western blot analysis. Cells were lysed in RIPA buffer (Cell Signaling) with complete protease inhibitor cocktail (Roche) and PMSF (Sigma). Protein lysates were quantified and resolved on an SDS-PAGE gel, transferred onto a PVDF membrane (Millipore) and immunoblotted with a primary antibody, followed by incubation with a secondary antibody. The antibody signal was detected using Amersham ECL Select Western blotting detection reagent (GE Healthcare). The following antibodies were used: anti-THBS2 (1:200, Santa Cruz, sc-12313), anti-β-actin (1:5000, Sigma-Aldrich, A5316), anti-H3K4me3 (1:1000; Abcam, ab8580), anti-H3K9ac (1:1000, Abcam, ab4441), anti-H3K9me3 (1:1000, Abcam, ab8898), anti-H3K27me3 (1:1000, Millipore, 07-449) and anti-histone H3 (1:1000, Abcam, ab24834).

Flow cytometry and cell sorting. Flow cytometry analysis and flow cytometry cell sorting was conducted using PE-conjugated anti-CD133 (Miltenyi Biotec), FITC-conjugated anti-EpCAM (BD Biosciences), FITC-conjugated anti-CD13 (eBiosciences), FITC-conjugated anti-CD44 (BD Biosciences), FITC-conjugated anti-CD90 (BD Biosciences), APC-conjugated anti-OV-6 (R&D Systems), anti-K19 (Abcam) and their respective isotype controls (eBiosciences). ALDEFLUOR^{TM} kit (STEMCELL Technologies) was used to measure ALDH activity. Samples were analyzed and sorted on a BD FACSCanto II and FACSAria I cytometers, respectively (BD Biosciences), and the data were analyzed by FlowJo software (Tree Star Inc.).

Immunohistochemistry assays. Slides were heated for antigen retrieval in 10 mM sodium citrate (pH 6.0). Endogenous peroxidase activity was inhibited with 3% hydrogen peroxide. Sections were subsequently incubated with anti-THBS2 (1:50, Santa Cruz, sc-12313), anti-CD133 (1:100, Millipore, MAB4310), anti-CD34 (1:500, BD Biosciences, 555820) and anti-cleaved caspase 3 (1:100, Cell Signaling Technology, 9661). The reaction was developed with a DAB+ substrate-chromogen system (Dako). Slides were counterstained with Mayer's hematoxylin. Serial sections were stained with Masson's trichrome stain (Sigma-Aldrich) and picrosirius red stain (Sigma-Aldrich) for collagen and fibrillar collagen, respectively. For TUNEL assay, apoptosis was detected using the *In Situ* Cell Death Detection Kit, POD (Sigma-Aldrich).

Lentiviral production and cell transduction. THBS2 (NM_003247) lentiviral overexpression and empty vector control plasmids (pReceiver-Lv105) were purchased from GeneCopoeia. Sequences were transfected into 293T cells and packaged using pMDLg/pRRE (Addgene 12251), pRSV-Rev (Addgene 12253) and pMD2.G (Addgene 12259). Expression plasmids for shRNAs were cloned into a pLKO.1-blast vector (Addgene 26655). The sequences of two shRNAs directed against THBS2 were clone ID NM_003247.2-1137s1c1 (5'-CCGGGTGTCGAATGATAACCAGTTTCTCGAGAAACTGGTTATCATTCGACACTTTTTG-3') and clone ID NM_003247.2-2606s1c1 (5'-CCGGCCAGATCGACACAGACAACAACTCGAGTTGTTGTCTGTGTCGATCTGGTTTTTG-3'). The target sequence for the scrambled shRNA nontarget control was NTC (5'-CCGGCAACAAGATGAAGAGCACAACTCGAGTTGGTGCTCTTCATCTTGTTGTTTTT-3'). Sequences were transfected into 293FT cells and packaged using MISSION Lentiviral Packaging Mix (Sigma-Aldrich). Virus-containing supernatants were collected for subsequent transduction to establish cells with stably overexpressed and repressed THBS2. Puromycin and blasticidin were used for cell selection.

Cell motility and invasion assays. Migration and invasion assays were conducted in 24-well Millicell hanging inserts (Millipore). For the migration assay, cells resuspended in serum-free DMEM were added to the top chamber, and medium supplemented with 10% FBS was added to the bottom chamber as a chemoattractant. For the invasion assay, inserts were precoated with diluted Matrigel (1:10) for 2 hrs

before the cell suspension was added. After 48 hrs of incubation at 37°C, the cells that migrated or invaded through the membrane (migration) or Matrigel (invasion) were fixed with 4% paraformaldehyde and stained with 20% crystal violet (Sigma-Aldrich). The number of cells was counted in 3 random fields under an inverted microscope (Eclipse Ts2; Nikon) equipped with a 4x objective lens.

Annexin V apoptosis assay. Cells were treated with the chemotherapeutic drug 5-fluorouracil for 2 days (200 µg/ml for HCC cell lines and 500 µg/ml for HCC organoids). Following treatment, the cells were harvested and stained with propidium iodide (PI) (BioLegend) and FITC-conjugated Annexin V (BioVision). The samples were analyzed on a BD FACSCanto II (BD Biosciences) with data analyzed by FlowJo (Tree Star Inc.).

***In vitro* limiting dilution assay (LDA).** Single cells were cultured at limiting dilutions in 100 µl of serum-free DMEM/F12 medium (Invitrogen) supplemented with 20 ng/ml human recombinant epidermal growth factor (Sigma-Aldrich), 10 ng/ml human recombinant basic fibroblast growth factor (Sigma-Aldrich), 4 µg/ml insulin (Sigma-Aldrich), B27 (1:50; Invitrogen), 500 U/ml penicillin, 500 µg/ml streptomycin (Invitrogen) and 1% methylcellulose (Sigma-Aldrich). The cells were cultured in suspension in poly-HEMA-coated 96-well plates. Wells were monitored for sphere formation at days 5-7. Stem cell frequency was calculated using extreme limiting dilution analysis (http://bioinf.wehi.edu.au/software/elda/) [55].

Gelatin degradation assay. Cells were plated on Oregon Green 488-gelatin (Invitrogen)-coated coverslips in 24-well plates and incubated for 72 hrs at 37°C. The cells were fixed with 4% paraformaldehyde, counterstained with anti-fade DAPI (Invitrogen) and visualized by confocal microscopy (Carl Zeiss LSM 700) equipped with Plan-Apochromat 40x/1.4 Oil DIC objective lenses. Foci of the degraded matrix appeared as dark areas that lacked fluorescence and were recognized as 'holes' in the bright fluorescent gelatin matrix. Images were captured and analyzed using ZEN 2010 Software (Zeiss Microscopy).

Gelatin zymography. MMP2 and MMP9 activity was measured by gelatin zymography. Confluent cells were incubated on 150-mm culture dishes for 48 hrs in serum-free conditions. Conditioned medium was concentrated with centrifugation via an Amicon Ultra-15 Centrifugal Filter Units (10 NMWL, kDa). Concentrated conditioned medium denatured in nonreducing sample buffer (62.5 mM Tris-HCl, pH 6.8; 10% glycerol; 2% SDS; and 0.0025% bromophenol blue, final concentration) was loaded onto Novex Zymogram gelatin gels (0.1%; Invitrogen). Following electrophoresis, the gels were washed in renaturing buffer and incubated in developing buffer for 16 hrs at 37°C. After Coomassie blue staining, the area of protease activity appeared as clear bands against a dark background.

ChIP-qPCR. Chromatin immunoprecipitation (ChIP) was performed using a Magna ChIP G Chromatin Immunoprecipitation Assay Kit (Millipore). MHCC97L cells from soft and stiff matrix gels were cross-linked in the presence of 1% formaldehyde at room temperature for 10 min, followed by cellular and nuclear lysis. The lysates were then sheared by a sonicator using a condition previously optimized to yield fragments in the sizes ranging from 200 to 1000 bp. Then, sheared cross-linked chromatin-containing DNA-protein complexes isolated from $1x10^6$ cell equivalents of lysate were subjected to immunoprecipitation by overnight incubation with either the target antibodies H3K4me3 (Abcam, ab8580), H3K9ac (Abcam, ab4441), H3K9me3 (Abcam, ab8898), and H3K27me3 (Millipore, 07-449) or the negative control, rabbit IgG (Bethyl Laboratories, P120-101). One percent of sheared cross-linked

chromatin in each sample was labeled as input DNA to normalize the amount of chromatin used for multiple samples in each IP experiment. Immunoprecipitated and eluted DNA was purified in columns and analyzed by qPCR with the following primers: PROM1 (forward 5’-CATATCGCAGCGGTTGTTCG-3’ and reverse 5’- AAGGCGACTGACCTTTCTGG-3’) or THBS2 (forward 5’-CCACAGAATGCGTGTTTCCTG-3’ and reverse 5’- CCAGTGAGTGTCACGGTCTT-3’).

SUPPLEMENTARY FIGURES AND FIGURE LEGENDS

Supplementary Figure S1. (Related to Figure 1)

Flow cytometry analysis for expression of known liver CSC marker following 5-FU treatment. Representative flow cytometry dot plots of EpCAM, CD13, CD44, CD90, oval 6 (OV-6), keratin 19 (K19) and aldehyde dehydrogenase (ALDH) expression or activity in HCC cells before and after 5-fluorouracil (5-FU) treatment. DMSO was used as a control.

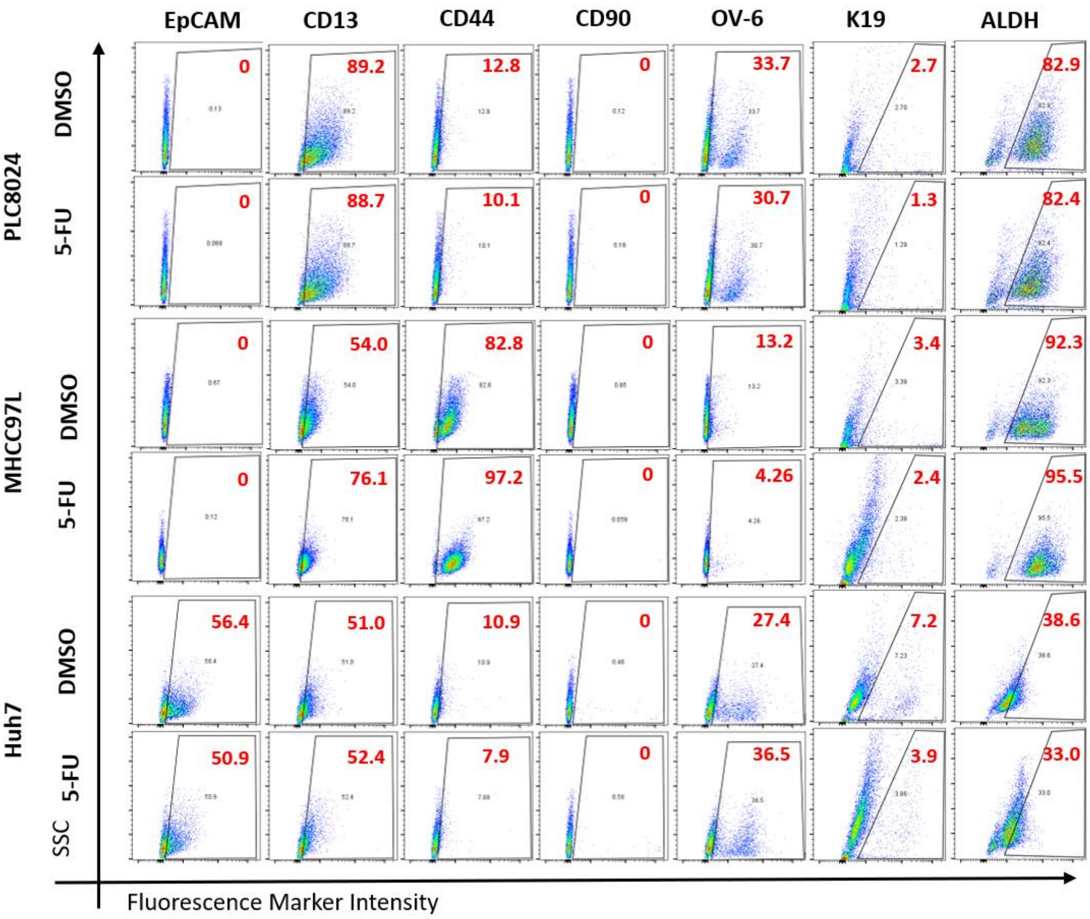

Supplementary Figure S2. (Related to Figure 1)

MMP2/MMP9 and THBS1 expression unchanged. (A) qPCR analysis of MMP2 and MMP9 expression in HCC cells with or without THBS2 expression modulated. **(B)** qPCR analysis for THBS1 expression in HCC cells with or without THBS2 expression modulated. EV for empty vector, OE for THBS2 overexpression, NTC for non-target control, 1137 and 2606 for two THBS2 shRNA knockdown clones.

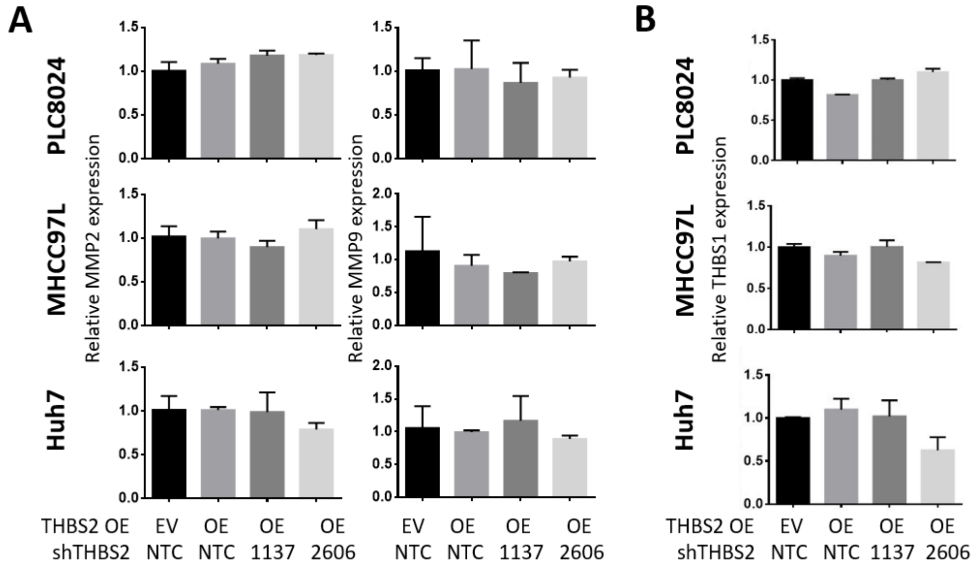

Supplementary Figure S3. (Related to Figures 2 and 3)

Functional THBS2 overexpression rescue in 5-FU treated HCC cells. Representative Annexin V apoptosis dot plots following treatment with 5-fluorouracil (5-FU) and with or without THBS2 overexpressed. DMSO was used as a control. EV for empty vector control and OE for THBS2 overexpression.

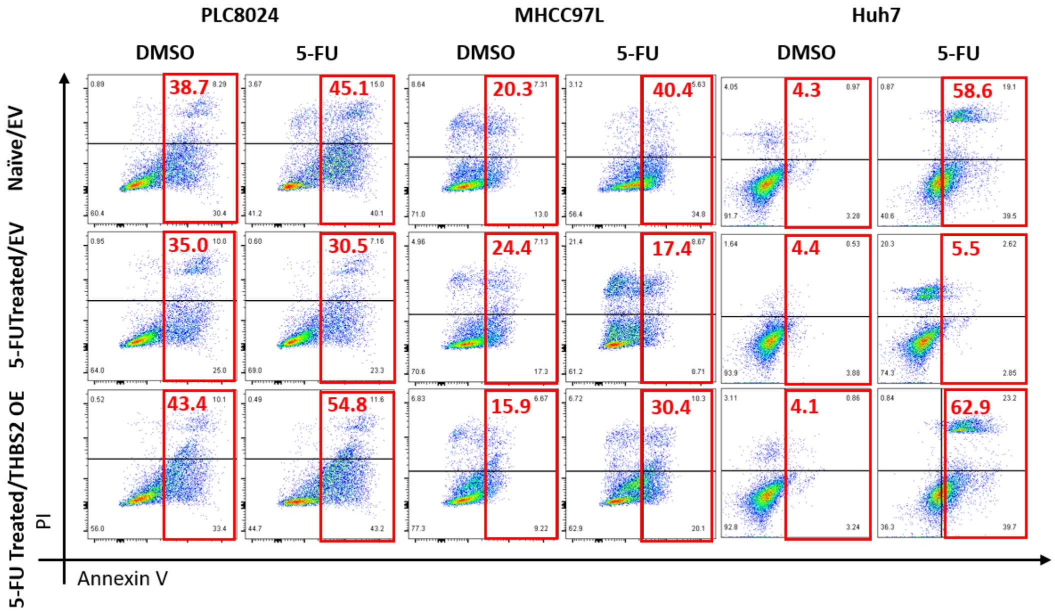

Supplementary Figure S4. (Related to Figures 2 and 3)

Recombinant THBS2 regulates cancer and stemness properties in HCC cells. HCC cells and patient-derived organoids with or without recombinant THBS2 (rTHBS2) treatment were grown in 3D matrix gel. PBS as control. **(A-B)** Representative images and quantification of number of cells that migrated (A) or invaded (B). **(C)** *In vitro* limiting dilution spheroid analysis. **(D)** Representative flow cytometry dot plots and quantification for CD133 expression in HCC cells treated with control or 5-FU. **(E)** Representative Annexin V apoptosis dot plots and quantification following treatment with 5-fluorouracil (5-FU). rTHBS2 for recombinant THBS2, 5-FU for 5-fluorouracil. Data expressed as the mean ± SEM; *p<0.05, **p<0.01 and ***p<0.001 from Student's t-test (A-B and E) or one-way ANOVA with Bonferroni's posttest (D) or chi-square test (C).

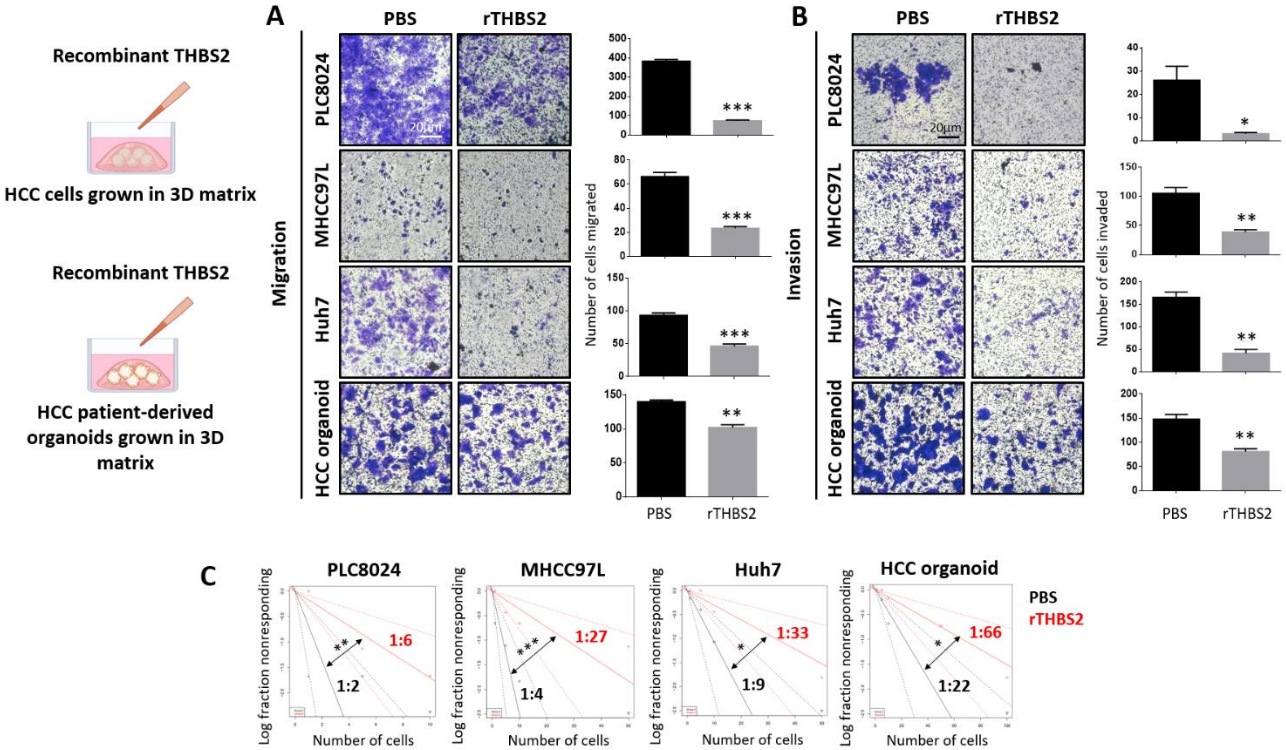

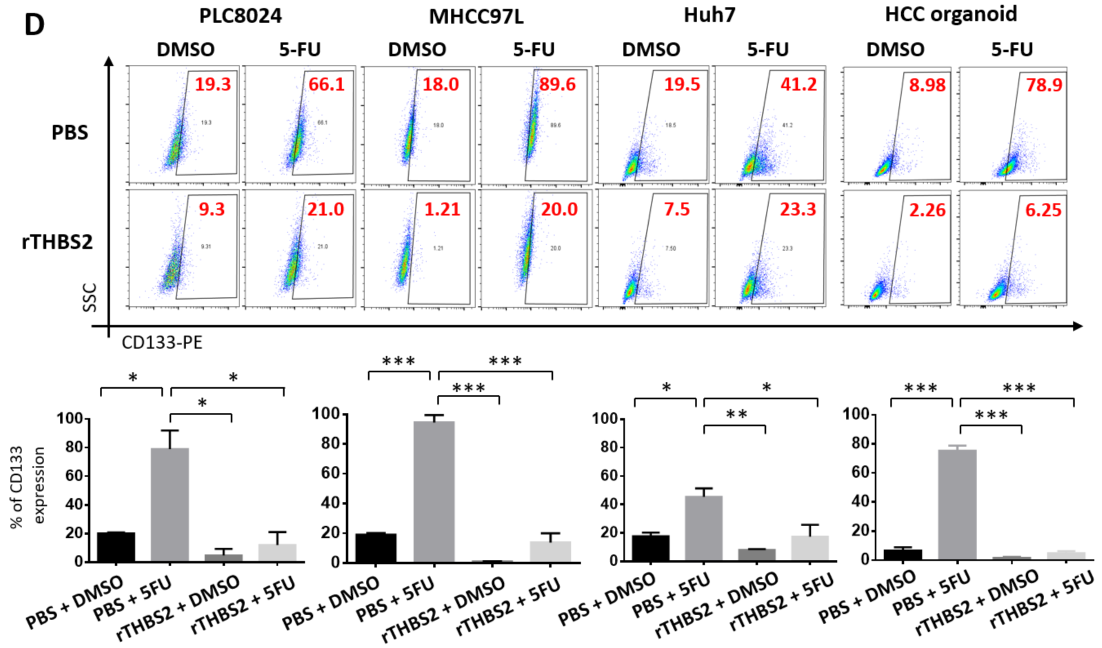
D
PLC8024
MHCC97L
Huh7
HCC organoid
DMSO
5-FU
PBS
rTHBS2
19.3
66.1
18.0
89.6
19.5
41.2
8.98
78.9
9.3
21.0
1.21
20.0
7.5
23.3
2.26
6.25
SSC
CD133-PE
% of CD133 expression
PBS + DMSO
PBS + 5FU
rTHBS2 + DMSO
rTHBS2 + 5FU

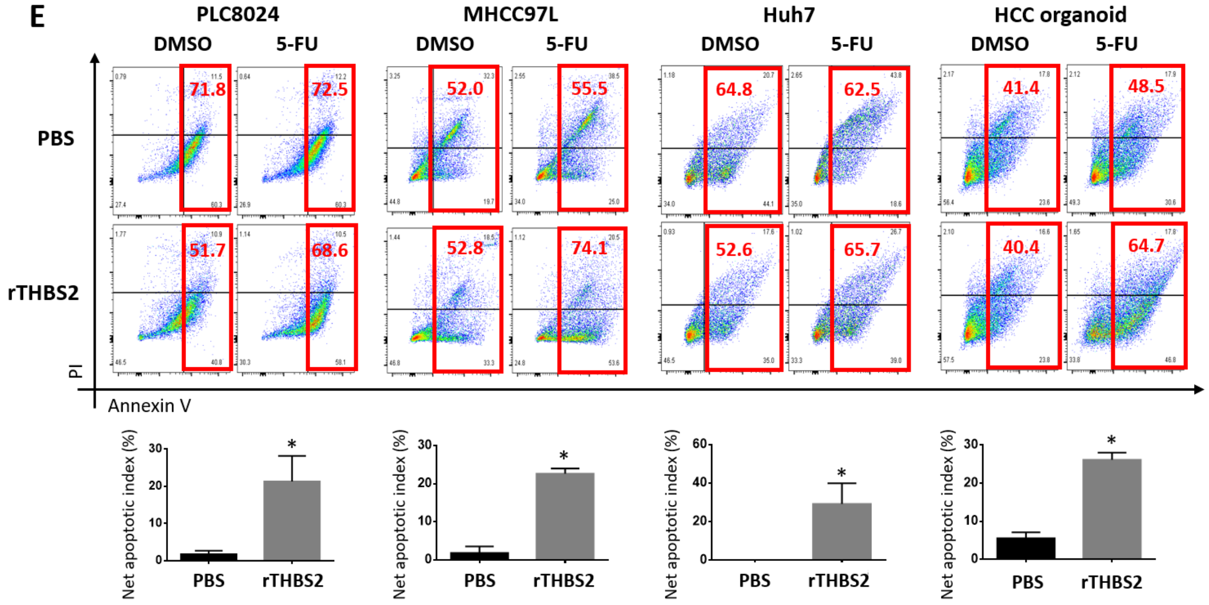
E
PLC8024
MHCC97L
Huh7
HCC organoid
DMSO
5-FU
PBS
rTHBS2
71.8
72.5
52.0
55.5
64.8
62.5
41.4
48.5
51.7
68.6
52.8
74.1
52.6
65.7
40.4
64.7
PI
Annexin V
Net apoptotic index (%)
PBS
rTHBS2

Supplementary Figure S5. (Related to Figure 2)

Recombinant THBS2 regulates cancer and stemness properties in HCC cells via matrix gel modification. HCC cells with or without recombinant THBS2 (rTHBS2) and/or ROCK inhibitor Y27632 treatment were grown on 2D plastic surface or in 3D matrix gel. **(A)** Representative images and quantification of number of cells that migrated or invaded. **(B)** *In vitro* limiting dilution spheroid analysis. **(C)** Representative Annexin V apoptosis dot plots and quantification following treatment with 5-fluorouracil (5-FU). rTHBS2 for recombinant THBS2, 5-FU for 5-fluorouracil and PI for propidium iodide. Data expressed as the mean ± SEM; *$p<0.05$, **$p<0.01$ and ***$p<0.001$ from one-way ANOVA with Bonferroni's posttest (A and C) or chi-square test (B).

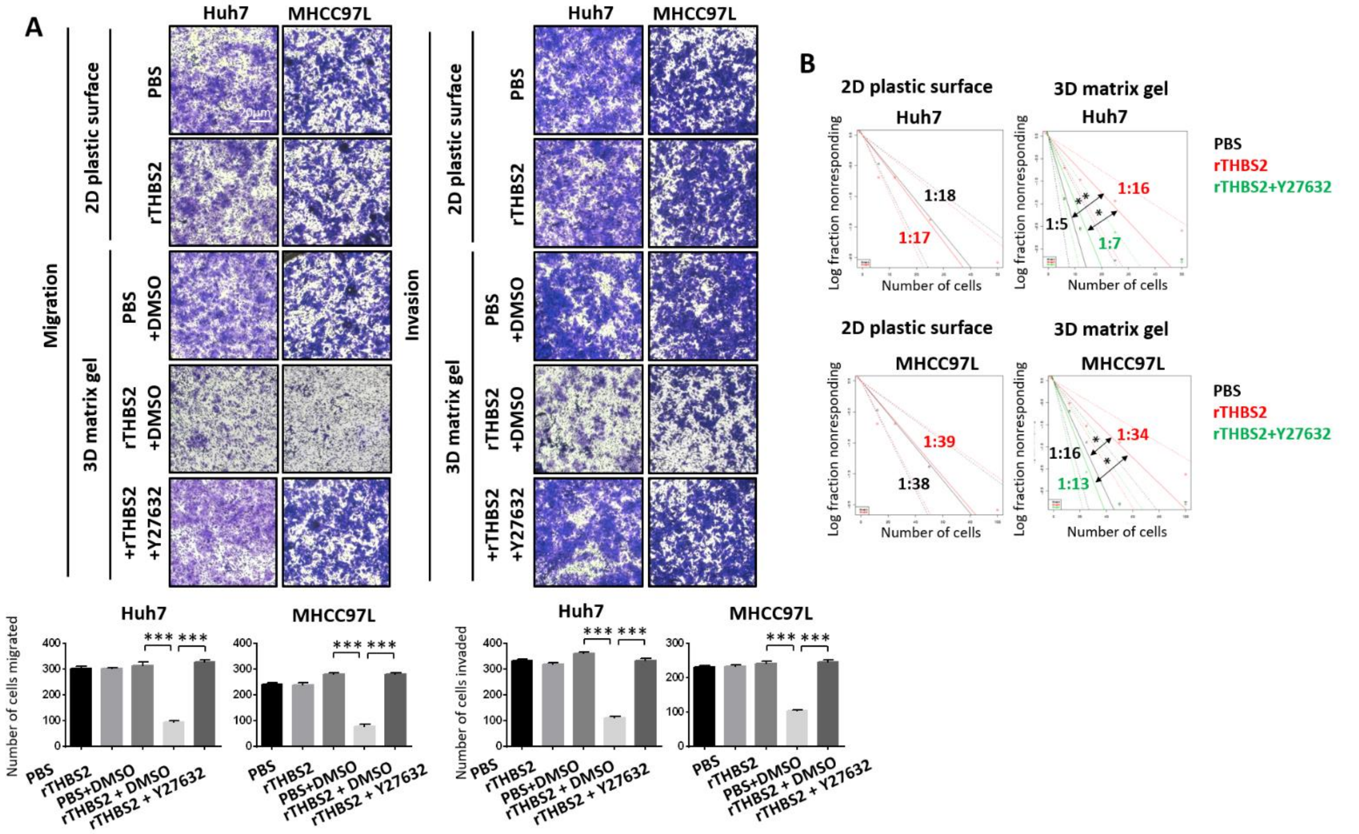

C

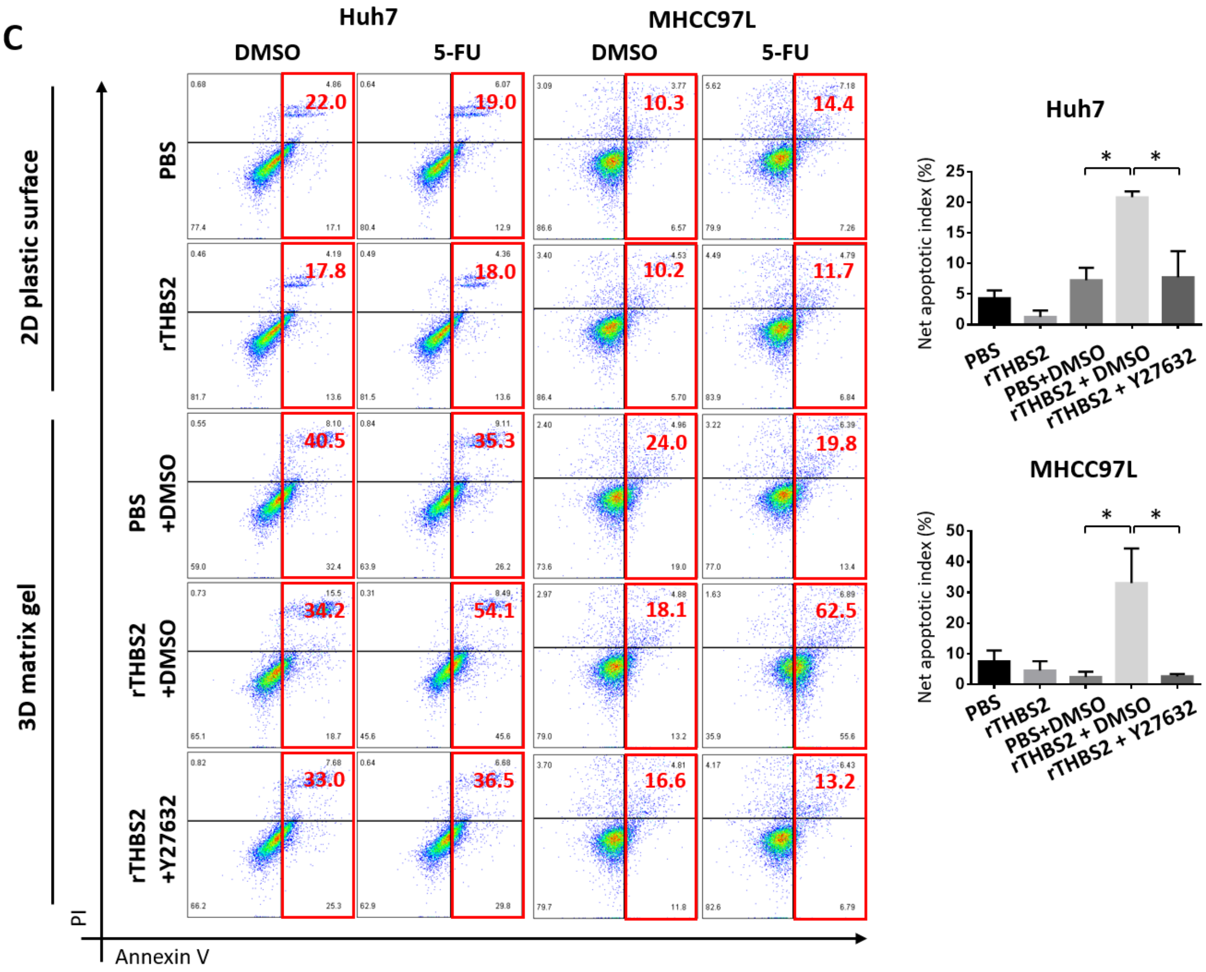
Huh7
MHCC97L
DMSO
5-FU
DMSO
5-FU
2D plastic surface
3D matrix gel
PBS
rTHBS2
PBS +DMSO
rTHBS2 +DMSO
rTHBS2 +Y27632
22.0
19.0
10.3
14.4
17.8
18.0
10.2
11.7
40.5
35.3
24.0
19.8
34.2
54.1
18.1
62.5
33.0
36.5
16.6
13.2
PI
Annexin V
Huh7
Net apoptotic index (%)
PBS
rTHBS2
PBS+DMSO
rTHBS2 + DMSO
rTHBS2 + Y27632
MHCC97L
Net apoptotic index (%)
PBS
rTHBS2
PBS+DMSO
rTHBS2 + DMSO
rTHBS2 + Y27632

Supplementary Figure S6. (Related to Figures 3, 4 and 5)

Quantification of collagen fiber thickness. Average thicken of collagen fiber (µm) in **(A)** livers resected from BALB/C nude mice intrahepatically injected with empty vector (EV) control or THBS2 overexpressing (OE) MHCC97L cells, **(B)** livers resected from BALB/C nude mice intrahepatically injected with MHCC97L HCC cells primed with recombinant THBS2 (rTHBS2) modified matrix gel, **(C)** livers resected from BALB/C nude mice intrahepatically injected with MHCC97L cells primed with or without recombinant THBS2 (rTHBS2) modified matrix gel and with or without 5-FU treatment and **(D)** human HCC clinical tissue samples that display high CD133 and low THBS2 (soft) or low/noCD133 and high THBS2 expression (stiff). EV for empty vector, OE for THBS2 overexpression, rTHBS2 for recombinant THBS2, 5-FU for 5-fluorouracil. Data expressed as the mean ± SEM; *$p<0.05$, **$p<0.01$ and ***$p<0.001$ from Student's *t*-test (A, B and D) or one-way ANOVA with Bonferroni's posttest (C).

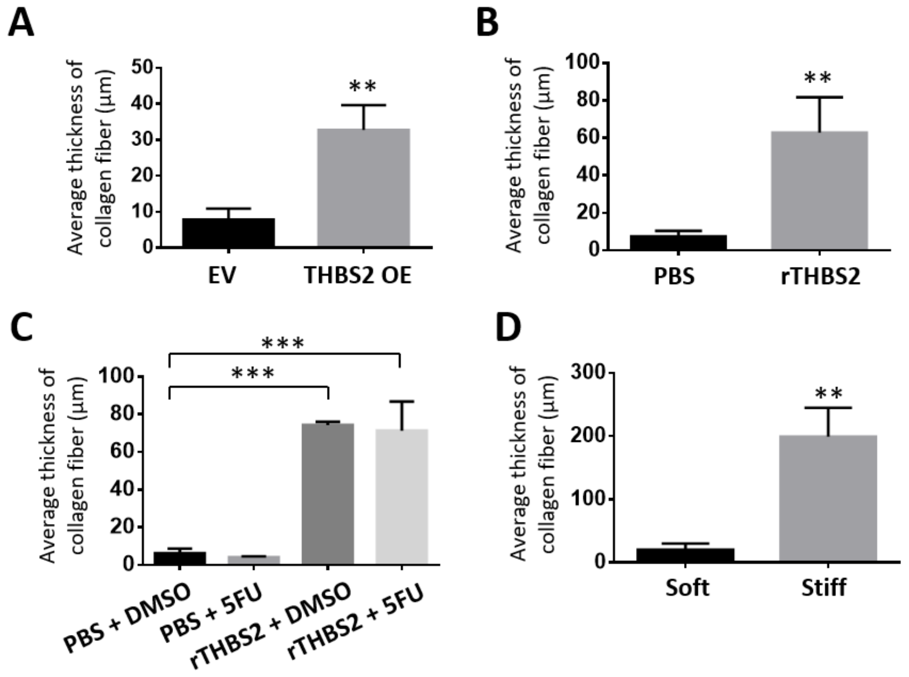

Supplementary Figure S7. (Related to Figure 3)

THBS2 attenuates metastatic abilities of HCC cells through modulating collagen degradation and tumor stiffness. (A) *In vivo* and *ex vivo* imaging for luciferase signal in BALB/C nude mice intrahepatically injected with 400,000 empty vector (EV) control or THBS2 overexpressing (OE) MHCC97L cells. Box plot shows the number of lung metastasis nodules recorded. *n* = 6-7 mice per group. **(B)** Representative H&E, Masson's trichrome and picrosirius red staining; and immunohistochemical staining for THBS2, CD133 and CD34 expression in serial sections of orthotopic liver xenograft tumors. Scale bar, 100μm. **(C)** Representative elastography images showing size of tumors (B-mode image analysis) and stiffness of tumor tissue (SWE image analysis). Scale bar, 2 mm. The mean elasticity values reflecting stiffness of the tumors are illustrated in the box plot (left). Box plots (right) show stiffness measurements of *ex vivo* livers harvested from above mouse model by indenter tests. EV for empty vector, OE for THBS2 overexpression, SWE for shear wave elastography. Data expressed as the mean ± SEM; *$p<0.05$, **$p<0.01$ and ***$p<0.001$ from Student's *t*-test.

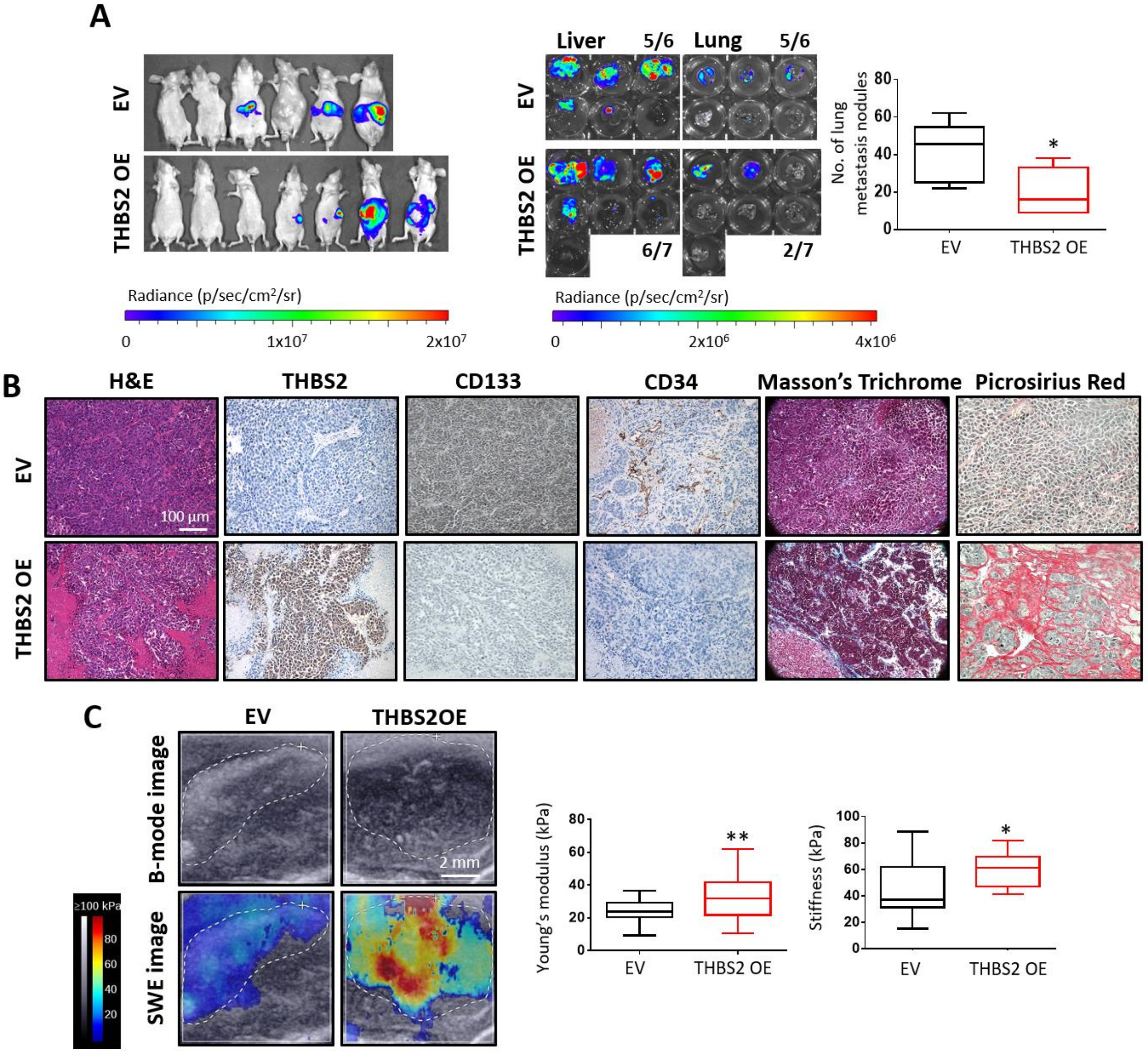

Supplementary Figure S8. (Related to Figure 5)

Immunohistochemistry analysis of THBS2 expression in clinical samples. Representative immunohistochemical analysis of no, weak or strong THBS2 expression in HCC tumor and adjacent nontumor liver tissue. Scale bar, 50 µm. Note since strong THBS2 was not detected in HCC, no representative image is shown.

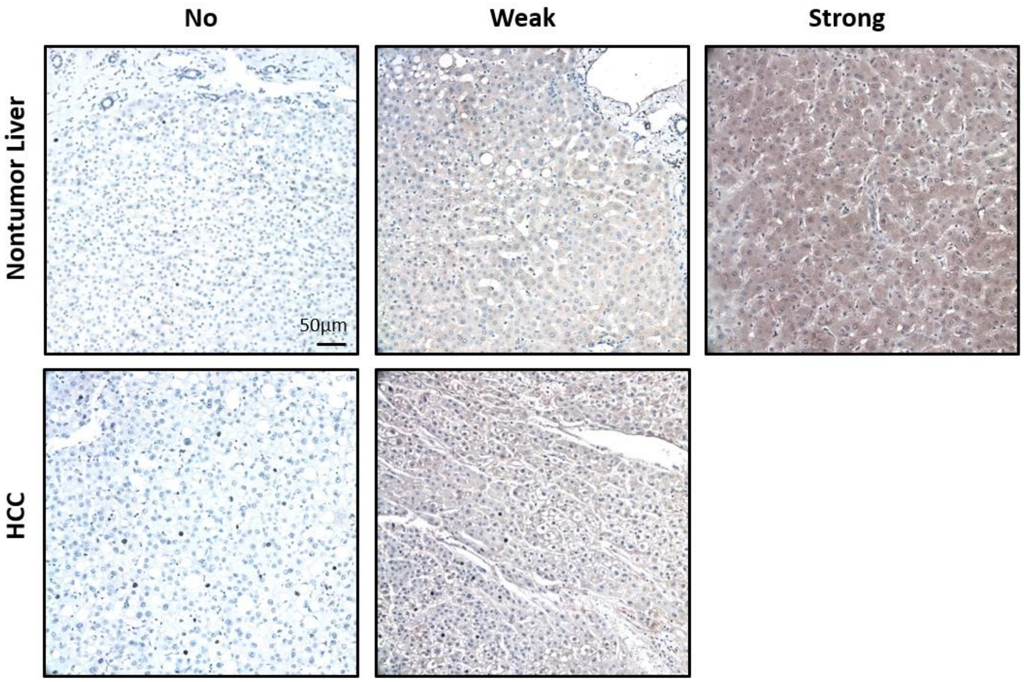

Supplementary Figure S9. (Related to Figure 6)

Promoter region of PROM1 is rich in transcriptional activating markers (H3K4me3 and H3K9ac) while that of THBS2 is rich in transcriptional silencing markers (H3K9me3 and H3K27me3). ChIP-seq data of HepG2 HCC cell was retrieved from ENCODE histone modification tracks by Broad Institute using Integrative Genomics Viewer. Activating markers H3K4me3 (green peaks) and H3K9ac (purple peaks) were found in PROM1 promoter region. Silencing markers H3K9me3 (blue peaks) and H3K27me3 (red peaks) were found in THBS2 promoter region.

PROM1 promoter

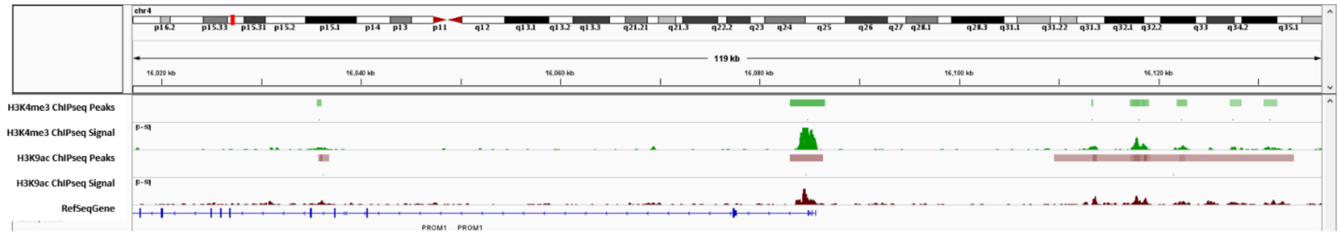

THBS2 promoter

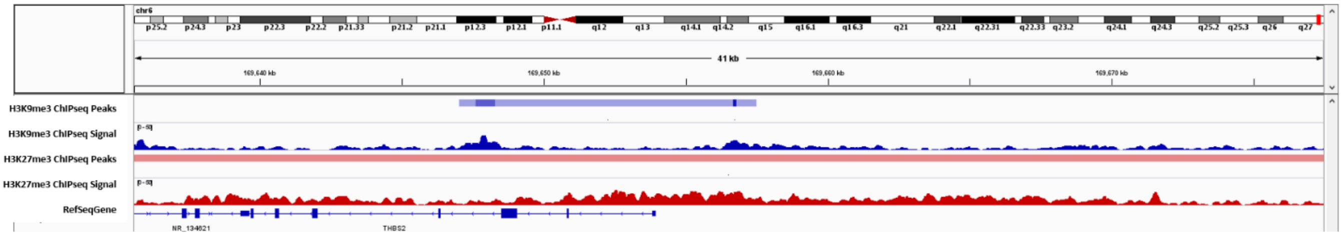

Supplement: Supplementary file 1 — Supporting Information [file ADVS-8-2002483-s001.pdf]
